# MF-YOLO: High-Resolution Weighted Feature Fusion with Global-Local Aggregation for Small-Object Detection in UAV Imagery

**DOI:** 10.3390/s26196084

**Published:** 2026-09-25

**Authors:** Yufei Wang, Jiming Yang, Fang Liu, Jun Liu

**Affiliations:** 1 School of Information Science and Engineering, Shenyang Ligong University, Shenyang 110159, China; 2 School of Computer Science and Engineering, Northeastern University, Shenyang 110169, China

**Keywords:** unmanned aerial vehicle, small-object detection, YOLO11, high-resolution prediction, weighted bidirectional feature fusion, global-to-local spatial aggregation

## Abstract

Small-object representations in unmanned aerial vehicle (UAV) imagery are easily degraded by repeated downsampling, crowded occlusion, and background clutter. We present MF-YOLO, an accuracy-oriented detector based on YOLO11n. It introduces a stride-4 P2 prediction scale, applies Global-to-Local Spatial Aggregation (GLSA) independently to C2–C5 before neck fusion, and connects P2–P5 through a 64-channel Bidirectional Feature Pyramid Network (BiFPN). The contribution lies in the task-specific organization and controlled evaluation of these established components. Across three random seeds, MF-YOLO achieved 38.318 ± 0.378% mean average precision at an intersection-over-union threshold of 0.5 (mAP@0.5) and 22.658 ± 0.239% mAP@0.5:0.95 on the validation split, exceeding YOLO11n by 4.264 and 2.694 percentage points, respectively. In COCO-style scale evaluation, average precision for small objects (${AP}_{S}$) increased from 8.700 ± 0.092% to 12.802 ± 0.115%, a relative gain of 47.2%. The parameter count decreased from 2.584 M to 2.244 M, whereas giga floating-point operations (GFLOPs) increased from 6.323 to 14.365. On an RTX 4080 SUPER, 32-bit floating-point (FP32) network-forward throughput decreased from 144.22 to 70.50 frames per second (FPS). MF-YOLO therefore improves small-object detection at the cost of substantially greater computation.

## 1. Introduction

Unmanned aerial vehicles (UAVs) combine high mobility, wide-area coverage, and relatively low data-acquisition costs, which has led to their widespread use in traffic management, emergency response, public safety, infrastructure inspection, and urban monitoring. Reliable object detection is fundamental to downstream tasks such as counting, tracking, and scene understanding. UAV imagery nevertheless remains challenging: many objects occupy only a few pixels; apparent object size varies with altitude and viewing angle; and dense layouts, occlusion, motion blur, uneven illumination, and structured urban backgrounds reduce foreground-background separability. VisDrone captures many of these difficulties, and recent studies show that missed detections and class confusion remain substantial [1,2,3,4].

Single-stage detectors predict object classes and bounding boxes directly from feature maps and are therefore attractive for UAV applications. The You Only Look Once (YOLO) family is widely used for this purpose, and YOLO11 provides compact variants together with a mature training and deployment framework [5,6,7,8]. We therefore selected YOLO11n as the baseline. Its standard detection head operates at P3/8, P4/16, and P5/32. After repeated downsampling, however, a small object may occupy only a few effective feature cells, causing edge and local-contrast information to be weakened before prediction, particularly in crowded scenes containing visually similar instances.

Feature Pyramid Networks (FPNs) transmit high-level semantic information through a top-down pathway, whereas Path Aggregation Networks (PANets) introduce a bottom-up pathway to shorten the flow of localization information [9,10]. Bidirectional Feature Pyramid Networks (BiFPNs) extend these designs with repeated top-down and bottom-up fusion and learnable normalized weights that regulate the contribution of features at different scales [11]. This form of fusion is particularly relevant to UAV imagery, in which shallow features retain edges and fine spatial detail but are easily affected by background texture, whereas deeper features provide stronger semantics at lower spatial resolution [12,13]. Global-to-Local Spatial Aggregation (GLSA) complements multi-scale fusion by combining local-detail modeling with global contextual information before features enter the neck [14]. Adaptive downsampling can further improve scale handling, but its location must be chosen carefully because excessive reduction in spatial resolution may discard the cues required to detect small objects.

MF-YOLO does not simply stack additional detection layers or attention modules. Instead, it determines where high-resolution spatial information, global-to-local context modeling, and cross-scale fusion should be introduced within the compact YOLO11n detector. The model retains C2 as the high-resolution source for P2 prediction, refines C2–C5 independently before neck fusion, and transfers spatial and semantic information across P2–P5 through a fixed-width weighted bidirectional path. P2 prediction, BiFPN-style fusion, and GLSA are all derived from prior work; accordingly, our contribution concerns their task-specific organization and controlled evaluation within YOLO11n rather than the individual components themselves.

The architectural contribution has three parts. First, MF-YOLO constructs a four-scale P2–P5 prediction path and fuses aligned 64-channel features using learnable nonnegative normalized weights. Second, independent GLSA modules refine C2–C5 at their native resolutions before the features enter the neck. Third, controlled P2-only and BiFPN-only ablations, paired A1–A2 experiments across three random seeds, scale-specific evaluation, and standardized complexity measurements were used to identify the benefits and costs of this organization.

The remainder of the paper is organized as follows. Section 2 reviews UAV small-object detection, high-resolution feature fusion, and global-to-local or adaptive feature processing. Section 3 describes the MF-YOLO architecture and training objective. Section 4 defines the dataset audit, implementation settings, metrics, and controlled experimental design. Section 5 reports the quantitative and qualitative results together with limitations. Section 6 concludes the study.

## 2. Related Work

### 2.1. Small-Object Detection in UAV Imagery

Recent UAV detectors seek to preserve spatial detail while limiting the cost of processing large aerial images. HSP-YOLOv8 introduces a high-resolution prediction branch and feature enhancement for aerial targets [15], while SOD-YOLO modifies YOLOv8 to strengthen small-object representation [16]. DroneNet, YOLOv8-lite, and TOE-YOLO emphasize compact or efficient UAV detection under pronounced scale variation conditions [17,18,19]. More recent YOLO11-based methods likewise explore dedicated small-object branches, multi-scale interaction, and attention-guided feature selection [20,21]. Despite their architectural differences, these studies consistently indicate that a standard three-scale head may be insufficient for extremely small targets.

Recent work also considers contextual modeling, crowded scenes, and operational cost. MFCI-YOLO combines multi-scale fusion with contextual information [22]; DSGF-Net integrates dense feature extraction with gated fusion [23]; and GA-YOLO combines lightweight feature extraction with attention for traffic monitoring [24]. DetailCaptureYOLO, DAU-YOLO, SMA-YOLO, and SPDC-YOLO investigate different combinations of detail preservation, attention, and feature fusion [25,26,27,28]. Feature-balanced pyramids and deeper networks designed for complex UAV scenes further show that small-object performance depends on coordinating shallow spatial detail with semantic context [29,30].

Results reported by different studies are not automatically comparable because they may use different YOLO baselines, input sizes, training schedules, augmentation policies, evaluation implementations, and hardware. Moreover, a single overall mAP value may obscure whether an improvement benefits the numerous small pedestrians and vehicles in VisDrone or only a subset of easier classes. We therefore combined harmonized baseline experiments with clearly identified literature-reported results and analyzed scale-specific AP, per-class performance, held-out test-dev performance, variability across random seeds, and computational cost.

### 2.2. High-Resolution Prediction and Weighted Multi-Scale Fusion

FPN and PANet implement top-down and bottom-up interactions between pyramid levels, respectively. Adding a P2 head to a single-stage detector retains a stride-4 feature map and provides more prediction locations for small objects, but it also substantially increases spatial computation. For a 640 × 640 input, a 160 × 160 P2 feature map contains four times as many spatial locations as an 80 × 80 P3 feature map. EfficientDet uses BiFPN to regulate feature fusion through repeated bidirectional connections and nonnegative normalized weights [11]. Weighted summation also avoids the channel expansion produced by concatenating every input feature.

MF-YOLO applies this weighted-fusion principle to a dedicated P2–P5 path in YOLO11n. The four backbone features are first aligned to a common channel width. Semantic information is then propagated from P5 to P2, followed by a bottom-up pass that returns the fused high-resolution representation from P2 to P5. P2 therefore contributes to both directions of the neck instead of serving only as an additional prediction output.

Feature compatibility is a central challenge in high-resolution fusion. P2 preserves edges and local contrast, but it carries weaker semantics and more background texture than P4 or P5. Repeated upsampling can spread ambiguous responses, whereas aggressive downsampling can remove the localization information introduced by P2. Learnable BiFPN weights regulate the contribution of aligned inputs but cannot compensate for intrinsically weak representations. High-resolution prediction, channel alignment, and bidirectional routing must therefore be considered jointly for UAV imagery [31,32,33,34,35].

### 2.3. Global-to-Local Spatial Aggregation and Selective Downsampling

Local convolution is well suited to edges, corners, and short-range patterns, whereas pooling- or attention-based context modeling helps distinguish objects from repetitive backgrounds. GLSA was introduced as a dual-branch local-global aggregation structure [14]. In MF-YOLO, GLSA is used as a pre-fusion refinement block: each C2–C5 feature is processed independently at its native resolution and mapped to the common channel width before entering the neck.

Adaptive downsampling assigns content-dependent importance to candidate sampling locations and may preserve discriminative responses more effectively than fixed downsampling. Lightweight adaptive weighted downsampling (LAWDS) is one such implementation [36]. Its effect nevertheless depends on placement: applying it near P2 may remove high-frequency cues needed for tiny objects, whereas applying it only at deeper levels may have limited influence. LAWDS is therefore evaluated as an optional ablation component rather than included in the final detector by default.

GLSA and adaptive downsampling address different aspects of representation. GLSA enriches local detail and contextual information without changing spatial resolution, whereas LAWDS determines which responses survive downsampling. Their effects need not be additive. We therefore adopted a staged design: establishing the P2–P5 weighted bidirectional fusion path, evaluating pre-fusion GLSA, and then testing LAWDS as an optional extension.

## 3. Proposed MF-YOLO Method

### 3.1. Baseline and Overall Architecture

Table 1 summarizes the main architectural distinctions between MF-YOLO and the closest related designs. MF-YOLO uses YOLO11n as the baseline. For a 640 × 640 input, the backbone produces C2, C3, C4, and C5 at strides 4, 8, 16, and 32, respectively. Independent GLSA modules refine these features before cross-scale fusion, after which all levels are aligned to 64 channels. A top-down path conveys deep semantic information toward P2, and a bottom-up path returns fused high-resolution information toward P5. The detection head receives $O_{2}$, $O_{3}$, $O_{4}$, and $O_{5}$ at strides 4, 8, 16, and 32. Thus, MF-YOLO integrates P2 into the complete bidirectional fusion path rather than simply appending an extra head. The overall architecture of MF-YOLO is shown in Figure 1.

$G_{2}\;-\;G_{5}$ denote backbone features after GLSA refinement. $T_{2}\;-{\;T}_{4}$ are the top-down fusion nodes, whereas $O_{2}\;-\;O_{5}$ are the four outputs supplied to the detection head.

Table 2 summarizes the structural differences between YOLO11n and MF-YOLO. The P2 path increases computation on high-resolution feature maps, whereas fixed 64-channel alignment reduces the number of learnable neck parameters. Consequently, MF-YOLO contains fewer parameters but requires more floating-point operations.

### 3.2. P2–P5 Weighted Bidirectional Fusion

Let $G_{i}$ denote the GLSA-refined feature at pyramid level $i\in \left\{2,3,4,5\right\}$. Each fusion node receives n feature tensors with identical spatial and channel dimensions. Each raw scalar weight $w_{i}$ is constrained by the rectified linear unit (ReLU) and normalized according to Equation (1).

(1) $${\tilde{w}}_{i}=\frac{ReLU(w_{i})}{\epsilon +\sum_{j=1}^{n}ReLU(w_{j})},\epsilon =10^{-4}$$

The constant $\varepsilon =10^{-4}$ prevents division by zero when all raw weights approach zero. The resulting coefficient ${\hat{w}}_{i}$ is used in the weighted aggregation defined in Equation (2).

(2) $$F\left(X_{1},\ldots ,X_{n}\right)=\sum_{i=1}^{n}{\tilde{w}}_{i}X_{i}.$$

In Equation (2), $X_{i}$ is the i-th aligned input feature. Every input is projected to 64 channels before fusion, so additive aggregation avoids the channel expansion caused by concatenation. The fusion weights are optimized jointly with the detector and determine the contribution of the lateral, resized, and previously fused features at each node.

The top-down pathway begins with $T_{5}=G_{5}$. At each lower pyramid level, the deeper feature is resized by nearest-neighbor upsampling, combined with the lateral GLSA feature at the same resolution, and refined by a C3k2 block, as defined in Equation (3).

(3) $$T_{5}={\;G}_{5},T_{i}={\;R}_{i}^{td}\left(F\left(G_{i},Up\left(T_{i+1}\right)\right)\right),i=4,3,2.$$

In Equation (3), $Up\left(\cdot \right)$ denotes nearest-neighbor upsampling and $R_{i}^{td}\left(\cdot \right)$ denotes the top-down C3k2 refinement block at level i. The output $O_{2}=T_{2}$ initializes the bottom-up pathway. At the P3 node, a stride-2 projection of the original $C_{2}$ feature supplies an additional high-resolution shortcut. Equation (4) defines the remaining bottom-up outputs.

(4) $$\begin{array}{c} O_{2}=T_{2},\\ O_{3}={\;R}_{3}^{bu}\left(F\left(Down\left(C_{2}\right),G_{3},T_{3}\right)\right),\\ O_{4}=R_{4}^{bu}\left(F\left(Down\left(O_{3}\right),G_{4},T_{4}\right)\right),\\ O_{5}={\;R}_{5}^{bu}\left(F\left(Down\left(O_{4}\right),G_{5}\right)\right). \end{array}$$

In Equation (4), $Down\left(\cdot \right)$ denotes a stride-2 convolution, and $R_{i}^{bu}\left(\cdot \right)$ denotes the bottom-up C3k2 refinement block at level i. This pathway carries high-resolution information from P2 toward the coarser levels while retaining the lateral and top-down features at each scale. The detection head receives $O_{2},O_{3},O_{4},O_{5}$.

### 3.3. Global-to-Local Spatial Aggregation

GLSA refines each backbone feature at its native spatial resolution before cross-scale fusion. For an input tensor $X\in R^{C\times H\times W}$, Equation (5) divides the channels evenly into a local subset $X_{l}$ and a global subset $X_{g}$. Both subsets retain the spatial size H×W. Two independent 1 × 1 projections then map the subsets to 64 channels, producing $Q_{l}$ and $Q_{g}$ as specified in Equation (6). The projections perform channel mapping only and use neither batch normalization nor an activation function.

(5) $$X_{l},X_{g}=Split(X),X_{l},X_{g}\in R^{\frac{C}{2}\times H\times W}$$

(6) $$Q_{l}={\;P}_{l}^{1\times 1}\left(X_{l}\right),Q_{g}=P_{g}^{1\times 1}\left(X_{g}\right),Q_{l},Q_{g}\in R^{64\times H\times W}.$$

In Equation (6), $P_{l}^{1\times 1}$ and $P_{g}^{1\times 1}$ denote the local and global projections. Both projected branches contain 64 channels, so the local operator, global operator, and final compression layer use a fixed internal width instead of inheriting the original C2–C5 channel dimensions.

The local branch preserves fine spatial structure through the gated residual output L in Equation (7). The symbol ⊙ denotes elementwise multiplication, and σ denotes the sigmoid function. The residual term retains $Q_{l}$ directly, while $R\left(Q_{l}\right)$ supplies a learned modulation signal.

Equations (8) and (9) follow the executed sequence of the local branch. Equation (8) describes the first two residual transformations, whereas Equation (9) gives the third transformation and the final local mapping.

(7) $$L=Q_{l}+Q_{l}⊙\sigma \left(R\left(Q_{l}\right)\right).$$

(8) $$Z_{1}=C_{1\times 1}^{ReLU}\left(Q_{l}\right),\;\;\;Z_{2}=Z_{1}+D_{3\times 3}^{ReLU,dw}\left(Z_{1}\right),\;\;\;Z_{3}=C_{1\times 1}^{ReLU}\left(Z_{2}\right).$$

(9) $$Z_{4}=Z_{3}+D_{3\times 3}^{ReLU,dw}\left(Z_{3}\right),\;\;\;Z_{5}=C_{1\times 1}^{SiLU}\left(Z_{4}\right),\;\;\;R\left(Q_{l}\right)=W_{1\times 1}^{ReLU}\left(Z_{5}+D_{3\times 3}^{ReLU,dw}\left(Z_{5}\right)\right).$$

In Equations (8) and (9), C with the ReLU or SiLU superscript denotes a 1 × 1 convolution-batch-normalization block followed by the indicated activation. D denotes a 3 × 3 depthwise convolution-batch-normalization block with 64 groups followed by ReLU. W denotes the final bias-free 1 × 1 convolution followed by ReLU, without batch normalization. In the implementation, conv1 and conv3 correspond to the ReLU-activated C operator; conv2, conv4, and conv6 correspond to D; conv5 corresponds to the SiLU-activated C operator; and conv7 corresponds to W. Every intermediate tensor has 64 channels and retains the input spatial resolution. In the third residual unit, the output of conv6 is added to Z5 before conv7 is applied, consistent with the forward implementation.

The global branch uses attention pooling to summarize long-range context. A 64-to-1 1 × 1 convolution produces a spatial attention mask, and Softmax normalizes it over the H×W positions. The weighted sum yields a 64-channel context vector from $Q_{g}$. A 64-to-128 1 × 1 convolution, LayerNorm, and ReLU transform this vector before a 128-to-64 projection generates the channel gate. These operations are formalized in Equations (11) and (12).

Equation (10) combines the local output L and global output G, whereas Equations (11) and (12) define the attention-pooling and context-gating operations used to obtain the global output.

(10) $$Y_{GLSA}={Conv}_{1\times 1}\left(\left[L,G\right]\right).$$

(11) $$M={Softmax}_{HW}\left(W_{1\times 1,\;64\to 1}\left(Q_{g}\right)\right),A\left(Q_{g}\right)=\sum_{i=1}^{HW}M_{i}{Q_{g}}_{i}.$$

(12) $$G=Q_{g}+Q_{g}⊙\sigma \left(W_{1\times 1,\;128\to 64}\left(ReLU\left(LN\left(W_{1\times 1,\;64\to 128}\left(A\left(Q_{g}\right)\right)\right)\right)\right)\right).$$

In Equation (11), Softmax normalizes the attention mask across all spatial positions, and the weighted sum forms a 64-channel context vector. Equation (12) expands this vector to 128 channels, applies LayerNorm and ReLU, projects it back to 64 channels, and passes the result through a sigmoid gate. Elementwise modulation of $Q_{g}$ followed by residual addition produces G.

In Equation (10), $\left[L,G\right]$ indicates channel-wise concatenation of the two branch outputs. Because L and G each contain 64 channels, concatenation produces a 128-channel tensor. The output ${Conv}_{1\times 1}$ is a 128-to-64 convolution-batch-normalization-SiLU block. Four independent GLSA modules process C2–C5, and each returns a 64-channel feature to the weighted bidirectional fusion path. The complete GLSA forward path is summarized in Figure 2.

### 3.4. Detection Objective

MF-YOLO retains the label-assignment strategy and decoupled detection head used by YOLO11n. The training objective combines bounding-box regression, classification, and distribution focal loss (DFL). The coefficients are fixed across all experiments, and the total loss is defined in Equation (13).

(13) $$L=7.5L_{box}+0.5L_{cls}+1.5L_{DFL}.$$

In Equation (13), the three terms denote the bounding-box regression loss, classification loss, and DFL, respectively. The regression term uses Complete Intersection over Union (CIoU), which accounts for box overlap, center-point distance, and aspect-ratio consistency [37]. DFL represents each box offset as a discrete probability distribution to improve localization precision [38]. A scale-adaptive EIoU-NWD objective, based on Efficient IoU [39] and normalized Wasserstein distance [40], was evaluated in the controlled B1 experiment. It did not improve either mAP@0.5 or mAP@0.5:0.95 with the same settings, so the final MF-YOLO configuration retains the CIoU-based objective in Equation (13).

## 4. Experimental Protocol

### 4.1. Dataset and Label Audit

VisDrone2019-DET contains 10 official categories: pedestrian, people, bicycle, car, van, truck, tricycle, awning-tricycle, bus, and motor [1]. We used the official VisDrone2019-DET training, validation, and test-dev splits. Original comma-separated annotations were converted to normalized YOLO bounding-box format. Objects marked as ignored or outside the evaluation scope were excluded from training, and every image–annotation pair was checked before model development. The data audit identified one anomalous bounding-box annotation. To preserve the official VisDrone2019-DET split and maintain reproducibility, this annotation was retained unchanged rather than corrected or deleted. No missing annotation files or corrupted images were found. We therefore treat the anomalous box as a label-quality limitation, not as an additional data-cleaning intervention.

Table 3 reports the audited split statistics. Following COCO conventions, small, medium, and large objects have areas below $32^{2}$ pixels, from $32^{2}$ to below $96^{2}$ pixels, and at least $96^{2}$ pixels, respectively. Small objects account for 60.5% of valid training boxes, 68.6% of validation boxes, and 67.7% of test-dev boxes. Figure 3 and Figure 4 illustrate the pronounced class imbalance and predominance of small instances.

### 4.2. Implementation Details

All models were trained with the same local Ultralytics 8.3.9 codebase using Python 3.11.15, PyTorch 2.5.1, and CUDA 12.4. Training was performed on an NVIDIA GeForce RTX 4090 GPU(NVIDIA Corporation, Santa Clara, CA, USA) with 24 GB of memory; network-forward throughput benchmarks were conducted separately on an RTX 4080 SUPER GPU(NVIDIA Corporation, Santa Clara, CA, USA), as stated in Section 5.7. Trainable layers were initialized from the corresponding pretrained weights. Custom modules, configuration files, and checkpoints were audited within the submitted code package.

All inputs were resized to 640 × 640. Each run used 150 epochs, a physical batch size of 8, two data-loader workers, stochastic gradient descent (SGD), and automatic mixed precision (AMP). The initial learning rate was 0.01, the final learning-rate multiplier was 0.01, momentum was 0.937, and weight decay was 0.0005. The first three epochs formed the warm-up stage. Color jitter, random translation and scaling, horizontal flipping, and mosaic augmentation followed, as detailed in Table 4; mosaic was disabled during the final 10 epochs. The checkpoint with the highest validation mAP@0.5:0.95 was retained.

All architectural configurations included in the architecture ablation (A0, P2-only, BiFPN-only, A1, A2, and A3) were first compared using seed 0. To assess the cross-seed stability of the GLSA contribution, A1 without GLSA and A2 with pre-fusion GLSA were additionally trained using seeds 1 and 2. The A1 and A2 experiments used identical data splits, 640 × 640 inputs, 150 epochs, batch size, SGD settings, pretrained initialization, data processing, and evaluation code.

### 4.3. Evaluation Metrics

Precision and recall are defined in Equation (14).

(14) $$P=\frac{TP}{TP+FP},\;R=\frac{TP}{TP+FN}\;,$$

TP, FP, and FN denote true positives, false positives, and false negatives, respectively. Precision is the proportion of predicted positives that are correct, whereas recall is the proportion of ground-truth objects that are detected. Average precision (AP) is the area under the interpolated precision–recall curve.

The primary validation results in Table 5, Table 6, Table 7, Table 8 and Table 9 were calculated with the Ultralytics 8.3.9 validation routine. mAP@0.5 averages AP across the 10 object categories at an intersection-over-union (IoU) threshold of 0.50, whereas mAP@0.5:0.95 averages AP over IoU thresholds from 0.50 to 0.95 in increments of 0.05. These metrics were used to summarize performance across the three matched random seeds.

For the scale-specific analysis, predictions from the same validation images and seed-matched checkpoints were evaluated separately with pycocotools COCOeval after the original VisDrone annotations had been converted to COCO format. Inference used 640 × 640 inputs, a confidence threshold of 0.001, an NMS IoU threshold of 0.70, and no more than 300 detections per image. In the conversion used in this study, a ground-truth or predicted box was excluded when at least 50% of its area was covered by a VisDrone ignored region.

COCOeval reports ${AP}_{S}$ (small-object AP), ${AP}_{M}$ (medium-object AP), and ${AP}_{L}$ (large-object AP) for bounding-box areas smaller than 322 pixels, from 322 to below 962 pixels, and at least 962 pixels, respectively. Box areas are measured in the original image coordinates. The evaluator uses IoU thresholds from 0.50 to 0.95 and maxDets = [1, 10, 300].

The Ultralytics validator and COCOeval are separate evaluation implementations, and the COCO-style analysis also includes explicit annotation conversion and ignored-region filtering. Their absolute AP@0.5:0.95 values are therefore not directly interchangeable. Model differences are compared within a single evaluation protocol.

### 4.4. Controlled Experimental Design

To isolate high-resolution prediction from weighted bidirectional fusion, we introduce two single-factor controls under the unified training protocol. A0 is the original YOLO11n. P2-only preserves the conventional baseline neck and adds only a P2 prediction branch. BiFPN-only retains the three P3–P5 prediction scales and introduces only weighted bidirectional fusion. A1 combines P2 prediction with a P2–P5 weighted bidirectional path but omits GLSA. A2 extends A1 by applying pre-fusion GLSA to C2–C5. All five configurations were compared with seed 0, while A1 and A2 were additionally trained with seeds 1 and 2.

LAWDS and the localization objective were examined separately from the main architectural comparison. A3 differs from A2 only in the deepest P4-to-P5 downsampling operation, where the original downsampling layer is replaced by LAWDS. B1 uses the same architecture and training settings as A2 but replaces CIoU with scale-adaptive EIoU-NWD. These two comparisons isolate the changes associated with LAWDS and the localization objective, respectively. After A2 was selected as the final configuration, the A0 and A2 checkpoints from all three seeds were evaluated on the held-out test-dev split without additional fine-tuning. The remaining training and evaluation settings were kept unchanged within each comparison.

## 5. Experimental Results and Analysis

### 5.1. Architecture Ablation Study

Table 5 reports the seed-0 component ablation with the same training and evaluation settings. P2-only retains the baseline neck and adds the high-resolution prediction branch, whereas BiFPN-only preserves the three P3–P5 prediction scales and introduces weighted bidirectional fusion. A1 combines the P2 prediction branch with the P2–P5 bidirectional fusion path, and A2 further applies GLSA to the C2–C5 backbone features before fusion. The results show that P2-only increases mAP@0.5:0.95 from 19.849% to 20.867%, indicating that the additional high-resolution prediction scale contributes directly to small-object detection. In contrast, BiFPN-only yields 18.363%, suggesting that weighted fusion alone does not improve the baseline in the current configuration. Combining P2 prediction and bidirectional fusion in A1 raises mAP@0.5:0.95 to 22.600%, while the addition of GLSA in A2 further increases it to 22.804%. These seed-0 results indicate that the main improvement arises from the joint use of high-resolution prediction and multi-scale fusion, whereas GLSA provides a smaller incremental gain. The stability of this incremental change is examined across multiple random seeds in the following section.

**Table 5:** Independent ablation of P2 prediction, weighted bidirectional fusion, and GLSA (seed 0).

Configuration	P2 Projections	Weighted Bidirectional Fusion	GLSA	P (%)	R (%)	mAP@0.5 (%)	mAP@0.5:0.95 (%)	Parameters (M)	GFLOPs
A0(YOLO11n)	No	No	No	45.471	33.277	33.897	19.849	2.584	6.323
P2-only	Yes	No	No	45.952	35.514	35.622	20.867	2.671	10.4
BiFPN-only	No	Yes	No	41.425	32.508	31.845	18.363	1.857	5.4
A1	Yes	Yes	No	48.112	37.659	38.129	22.600	2.048	12.6
A2	Yes	Yes	Yes	47.630	37.814	38.490	22.804	2.244	14.365

Adding P2 alone increased mAP@0.5:0.95 from 19.849% to 20.867%, whereas BiFPN-only reduced it to 18.363%. Combining the P2 branch with weighted bidirectional fusion in A1 raised mAP@0.5:0.95 to 22.600%, which is 1.733 percentage points above P2-only and 4.237 percentage points above BiFPN-only.

For seed 0, the P2 branch provides the main individual improvement. Weighted bidirectional fusion does not improve the baseline on its own, but the stronger A1 result indicates that it becomes useful when paired with P2 prediction. Table 6 examines whether the smaller gain from GLSA is consistent across matched random seeds.

**Table 6:** Paired A1–A2 comparison across three random seeds.

Model	Seed	P (%)	R (%)	mAP@0.5 (%)	mAP@0.5:0.95 (%)
A1	0	48.112	37.659	38.129	22.600
A1	1	46.681	37.606	37.614	22.394
A1	2	48.835	36.892	37.830	22.347
A2	0	47.630	37.814	38.490	22.804
A2	1	48.482	37.945	38.579	22.788
A2	2	47.814	37.308	37.884	22.382
A1	Mean ± SD	47.876 ± 1.096	37.386 ± 0.428	37.858 ± 0.259	22.447 ± 0.135
A2	Mean ± SD	47.975 ± 0.448	37.689 ± 0.336	38.318 ± 0.378	22.658 ± 0.239
A2–A1	Mean paired difference ± SD	+0.099 ± 1.498	+0.303 ± 0.134	+0.460 ± 0.463	+0.211 ± 0.180

Across seeds 0, 1, and 2, A2 exceeded A1 in mAP@0.5:0.95 by 0.204, 0.394, and 0.035 percentage points. The mean paired gain was 0.211 ± 0.180 percentage points, with a 95% confidence interval of [−0.235, 0.657]. The change was positive in every run, although the interval includes zero. GLSA is therefore treated as a modest refinement rather than a statistically established source of improvement. Table 7 places this comparison within the complete seed-0 architecture sequence, including the LAWDS extension A3.

**Table 7:** Seed-0 architecture ablation on the validation split.

ID	P2-BiFPN	GLSA at C2–C5	Selective LAWDS	P (%)	R (%)	mAP@0.5 (%)	mAP@0.5:0.95 (%)
A0	No	No	No	45.471	33.277	33.897	19.849
A1	Yes	No	No	48.112	37.659	38.129	22.600
A2	Yes	Yes	No	47.630	37.814	38.490	22.804
A3	Yes	Yes	Yes	48.929	37.035	38.238	22.821

A3 increased mAP@0.5:0.95 by only 0.017 percentage points relative to A2, while recall and mAP@0.5 decreased by 0.779 and 0.252 percentage points. This additional downsampling change did not improve the main accuracy measures consistently. A2 was retained because it provides a better balance of recall, mAP@0.5, architectural simplicity, and computational cost.

The validation trajectories in Figure 5 support this interpretation. A1–A3 separate from A0 early in training and converge to similar values after approximately 100 epochs. At seed 0, A2 attains the highest mAP@0.5, while the final mAP@0.5:0.95 curves of A2 and A3 are nearly indistinguishable.

Figure 6 compares the seed-0 precision, recall, mAP@0.5, and mAP@0.5:0.95 values. A2 achieves 47.630%, 37.814%, 38.490%, and 22.804%, respectively. A3 further increases precision but does not improve the other metrics consistently. Considering the reported detection metrics together with architectural simplicity and computational cost, A2 was selected as the final MF-YOLO configuration.

### 5.2. Loss-Function Analysis

Table 8 compares two localization objectives while keeping the A2 architecture fixed. With CIoU, A2 obtains 47.630% precision, 37.814% recall, 38.490% mAP@0.5, and 22.804% mAP@0.5:0.95. B1 replaces only CIoU with scale-adaptive EIoU-NWD and obtains 48.602%, 37.551%, 38.430%, and 22.531%, respectively. Thus, B1 increases precision by 0.972 percentage points but decreases recall, mAP@0.5, and mAP@0.5:0.95 by 0.263, 0.060, and 0.273 percentage points, respectively. CIoU was therefore retained in the final A2 model.

**Table 8:** Controlled localization-loss ablation for A2 with seed 0.

Configuration	Localization Objective	P (%)	R (%)	mAP@0.5 (%)	mAP@0.5:0.95 (%)
A2	CIoU	47.630	37.814	38.490	22.804
B1	Scale-adaptive EIoU-NWD	48.602	37.551	38.430	22.531

Changing the localization objective mainly shifted the precision–recall balance. The higher precision of B1 was accompanied by lower recall and lower AP, suggesting that greater emphasis on normalized center and size similarity did not resolve the dominant errors in dense UAV scenes. The final model therefore retains CIoU.

### 5.3. Repeatability Across Random Seeds

To assess whether the improvement over YOLO11n was consistent across training runs, the selected A2 configuration and the baseline were trained with three matched random seeds. Table 9 reports the resulting validation metrics obtained using the Ultralytics 8.3.9 routine.

**Table 9:** Three-seed repeatability on the validation split.

Model	Seed	P (%)	R (%)	mAP@0.5 (%)	mAP@0.5:0.95 (%)
YOLO11n	0	45.471	33.277	33.897	19.849
YOLO11n	1	45.586	33.472	34.110	20.063
YOLO11n	2	45.673	33.658	34.154	19.981
YOLO11n	Mean ± SD	45.577 ± 0.101	33.469 ± 0.191	34.054 ± 0.137	19.964 ± 0.108
MF-YOLO	0	47.630	37.814	38.490	22.804
MF-YOLO	1	48.482	37.945	38.579	22.788
MF-YOLO	2	47.814	37.308	37.884	22.382
MF-YOLO	Mean ± SD	47.975 ± 0.448	37.689 ± 0.336	38.318 ± 0.378	22.658 ± 0.239
MF-YOLO-YOLO11n	Mean paired difference ± SD	+2.399 ± 0.431	+4.220 ± 0.495	+4.264 ± 0.467	+2.694 ± 0.278

Table 9 summarizes independent YOLO11n and MF-YOLO runs using the same three random seeds. MF-YOLO increases the mean mAP@0.5 from 34.054% to 38.318% and the mean mAP@0.5:0.95 from 19.964% to 22.658%, corresponding to gains of 4.264 and 2.694 percentage points, respectively. Relative to the YOLO11n means, these changes represent improvements of approximately 12.5% and 13.5%. Pairing runs initialized with the same seed provides a direct comparison while reducing the influence of seed-dependent training variation. The paired gains in precision, recall, mAP@0.5, and mAP@0.5:0.95 were +2.399 ± 0.431, +4.220 ± 0.495, +4.264 ± 0.467, and +2.694 ± 0.278 percentage points, respectively. For seeds 0, 1, and 2, the mAP@0.5 gains were +4.593, +4.469, and +3.730 percentage points, while the corresponding mAP@0.5:0.95 gains were +2.955, +2.725, and +2.401 percentage points. Thus, every seed produced improvements in both metrics. MF-YOLO exhibits slightly greater dispersion than YOLO11n, particularly in mAP@0.5, but its lowest seed-specific result still exceeds the corresponding YOLO11n result. The observed advantage is therefore consistent across the three matched runs rather than being determined by a single favorable initialization. Given the limited number of seeds, these results are interpreted as evidence of cross-seed consistency rather than a formal claim of statistical significance.

Figure 7 presents the class-wise precision–recall behavior of the YOLO11n baseline. Car achieves the highest AP@0.5 at 77.6%, followed by bus at 57.2%, whereas bicycle and awning-tricycle obtain only 20.3% and 19.4%, respectively. The curves for these difficult categories decline rapidly as recall increases, showing that additional detections are accompanied by a substantial loss of precision. Pedestrian, people, van, truck, tricycle, and motor show intermediate AP@0.5 values, although their curves also reveal considerable room for improvement. This uneven class-wise performance is consistent with the challenges of VisDrone imagery, in which object scale, occlusion, instance density, and visual similarity vary markedly among categories. The baseline curves therefore provide a class-level reference for determining whether MF-YOLO produces broadly distributed improvements or benefits only the dominant categories.

Figure 8 shows the corresponding MF-YOLO results. AP@0.5 is higher for nine of the ten categories, with the largest gains observed for pedestrian, bus, and motor. Awning-tricycle is the only category with a slight decrease, indicating that the improvement is broad but not uniform across classes.

### 5.4. Held-Out Test-Dev Evaluation

After architecture selection, the three A0 and A2 checkpoints were evaluated on the held-out test-dev split without fine-tuning. Table 10 reports the mean and sample standard deviation. Although the absolute scores are lower than those on the validation split, MF-YOLO improves precision, recall, mAP@0.5, and mAP@0.5:0.95 by 1.996, 2.427, 2.856, and 1.444 percentage points, respectively.

The smaller gain on test-dev shows that the size of the improvement varies with the evaluation distribution. All four metrics nevertheless remain higher for MF-YOLO, so the direction of the effect is consistent across the validation and test-dev splits.

### 5.5. Scale-Specific and Per-Class Performance

Table 11 reports COCO-style AP after ignored-region handling. MF-YOLO increases ${AP}_{S}$ from 8.700% to 12.802%, an absolute gain of 4.102 percentage points and a relative gain of 47.2%. ${AP}_{M}$ increases from 26.950% to 29.304%, whereas ${AP}_{L}$ decreases from 44.697% to 36.507%, a reduction of 8.190 percentage points. Figure 9 visualizes the three scale-specific changes, and Table 12 presents the component-level scale analysis, while Table 13 reports per-class AP@0.5:0.95.

Across three seeds, the absolute changes in ${AP}_{S}$, ${AP}_{M}$, and ${AP}_{L}$ were +4.102, +2.354, and −8.190 percentage points, respectively. MF-YOLO therefore shifts the scale-performance balance toward small and medium objects rather than improving all object sizes.

Data imbalance is relevant to this trade-off: large objects account for only 5.53% of valid training labels and 2.76% of validation labels. The P2 prediction path and four-scale fusion strengthen local detail, while fixed 64-channel alignment and repeated cross-scale aggregation may constrain the P5 representation of complete contours and global semantics. This mechanism is an architectural interpretation rather than a proven single cause because the effects of data scarcity, channel alignment, and fusion routing are not isolated here.

Table 12 separates the scale effects of the architectural components using the best seed-0 checkpoints for A0, P2-only, BiFPN-only, A1, and A2, all evaluated with the same COCO-style procedure.

Relative to A0, P2-only changes ${AP}_{S}$, ${AP}_{M}$, and ${AP}_{L}$ by +2.521, −0.141, and −6.321 percentage points; BiFPN-only changes them by −1.194, −1.847, and −6.404 percentage points. A1 changes the three metrics by +3.930, +2.184, and −6.222 percentage points. Adding GLSA to A1 produces small positive changes of +0.238, +0.111, and +0.376 percentage points. Overall, A2 exceeds A0 by 4.168 percentage points in ${AP}_{S}$ and 2.295 percentage points in ${AP}_{M}$ while remaining 5.846 percentage points lower in ${AP}_{L}$.

These measurements identify P2 as the main source of the small-object gain. P2-only raises ${AP}_{S}$, whereas BiFPN-only does not improve any of the three scale metrics. The stronger A1 result shows that weighted bidirectional fusion becomes useful when it is coupled with the P2 branch, and GLSA then adds smaller gains to A1 at all three scales. Because Table 12 is based on seed 0, the comparison describes this experimental setting rather than a general significance claim.

Figure 9 summarizes the scale trade-off: the gains for small and medium objects are accompanied by lower AP for large objects. The corresponding three-seed means and standard deviations are reported in Table 11.

Table 13 and Figure 10 show that MF-YOLO improves AP@0.5:0.95 for eight of the 10 classes. The largest gains occur for pedestrian (+5.289 percentage points), car (+5.045), motor (+4.824), people (+4.563), van (+4.453), and bus (+3.360). Performance decreases slightly for truck (−0.516 percentage points) and awning-tricycle (−0.281 percentage points). These results indicate that the overall improvement is distributed across several object categories rather than being dominated by a single class. The small declines for truck and awning-tricycle also show that the proposed feature-fusion strategy does not benefit every category equally, which remains a limitation of the current model.

### 5.6. Comparison with Recent Representative Methods

Two comparison settings are reported. Table 14 contains models retrained in this study with the same VisDrone2019-DET split, 640 × 640 input, 150-epoch schedule, batch size, optimizer, data pipeline, and evaluation code. Table 15 lists published results for HSP-YOLOv8, SOD-YOLO-n, TOE-YOLO, and SRTSOD-YOLO-n on VisDrone2019 at 640 × 640. The latter provides context rather than a controlled ranking because training and evaluation details differ across studies.

Under the harmonized protocol, MF-YOLO achieves the highest precision (47.630%), recall (37.814%), mAP@0.5 (38.490%), and mAP@0.5:0.95 (22.804%) among the four models. Its mAP@0.5:0.95 exceeds YOLOv8n, YOLOv10n, and YOLO11n by 3.159, 2.273, and 2.955 percentage points, respectively. MF-YOLO also has the lowest parameter count (2.244 M), but its computational cost is 14.365 GFLOPs, compared with 7.0, 8.4, and 6.323 GFLOPs for the three baselines. The accuracy gain therefore comes with higher computational cost.

Table 15 reports the values published by the respective authors. Matching dataset names and input sizes do not ensure full comparability because data splits, training schedules, pretrained initialization, optimizers, random seeds, and evaluation implementations may differ.

The literature-reported results place MF-YOLO above SOD-YOLO-n, TOE-YOLO, and SRTSOD-YOLO-n in mAP@0.5 and mAP@0.5:0.95, but below HSP-YOLOv8. MF-YOLO has fewer parameters than HSP-YOLOv8, TOE-YOLO, and SRTSOD-YOLO-n but more than SOD-YOLO-n. Its GFLOPs are lower than HSP-YOLOv8 but higher than the other three lightweight methods. MF-YOLO therefore represents a specific trade-off among accuracy, parameter count, and computation rather than dominating every metric.

### 5.7. Complexity and Network-Forward Throughput Analysis

Table 16 reports parameter count, GFLOPs, median network-forward latency, network-forward throughput in frames per second (FPS), and peak GPU memory for YOLO11n and MF-YOLO for one controlled hardware benchmark. Measurements were obtained on an RTX 4080 SUPER with 640 × 640 inputs, batch size 1, 50 warm-up iterations, 300 timed iterations, and CUDA synchronization.

In FP32, MF-YOLO reduces the parameter count from 2.584 M to 2.244 M (13.2%) but increases GFLOPs from 6.323 to 14.365 (127.2%), or 2.27 times the baseline. Median network-forward latency rises from 6.934 to 14.184 ms, while network-forward throughput falls from 144.22 to 70.50 FPS. Fixed-width alignment reduces the number of learnable parameters, but computation increases because the P2 branch and the full P2–P5 fusion path operate on high-resolution features.

### 5.8. Qualitative Detection Results

Figure 11 compares YOLO11n and MF-YOLO predictions at seed 0 using a confidence threshold of 0.25. A prediction is counted as a true positive when it matches a ground-truth box of the same class at IoU ≥ 0.5. Across the two scenes, false negatives decrease from 135 to 109 and from 122 to 97, while false positives increase from 19 to 25 and from 20 to 37. The examples therefore illustrate improved recovery of distant vehicles and pedestrians together with a reduced precision margin in cluttered regions. In these examples, most additional false positives occur in densely packed areas where adjacent objects overlap or have weak visual boundaries. This behavior is consistent with the higher recall reported in Table 9, but it also indicates that retaining more small-object candidates can increase classification and localization errors in cluttered regions.

Remaining misses concentrate on heavily occluded pedestrian groups and extremely small objects close to the annotation limit. False positives often occur on repetitive roadside textures or between adjacent pedestrians. These cases suggest that future work should emphasize crowded-scene assignment and suppression strategies rather than simply adding further generic feature modules.

### 5.9. Diagnostic Visualizations

Diagnostic plots examine the accuracy–complexity trade-off, class confusion, and changes in feature responses. These comparisons use the same data split, checkpoint-selection rule, and decision settings.

Figure 12 places the five seed-0 configurations on the same accuracy–complexity plane. P2-only improves mAP@0.5:0.95 by 1.018 percentage points over A0, with GFLOPs increasing from 6.323 to 10.400. BiFPN-only requires 5.400 GFLOPs but also lowers mAP@0.5:0.95 to 18.363%. When P2 prediction and weighted bidirectional fusion are combined, A1 reaches 22.600%. Adding GLSA increases mAP@0.5:0.95 by a further 0.204 percentage points, yielding A2. A2 records the highest validation mAP@0.5:0.95 among the tested configurations, but it also has the largest computational requirement at 14.365 GFLOPs. The comparison therefore presents an accuracy–computation trade-off rather than an improvement in computational efficiency.

Figure 13 compares YOLO11n and MF-YOLO with the same hardware and timing procedure. MF-YOLO improves seed-0 validation mAP@0.5:0.95 by 2.955 percentage points, from 19.849% to 22.804%, while FP32 network-forward throughput decreases by 73.72 FPS, from 144.22 to 70.50 FPS. These measurements cover the model forward pass only and exclude image decoding, resizing, host-to-device transfer, and non-maximum suppression. The result confirms that the accuracy gain is accompanied by a substantial loss of throughput; it should not be interpreted as an end-to-end application speed measurement.

Figure 14 establishes the baseline error pattern. Missed detections are prominent for several small or visually ambiguous categories: 67% of pedestrian instances, 74% of people instances, and 82% of bicycle instances are assigned to background. Car has the strongest diagonal response among the foreground classes (0.72), although 40% of van instances are predicted as car. The matrix therefore shows that missed small objects and confusion among visually related road-user categories remain the main weaknesses of YOLO11n on this validation split.

Compared with Figure 14, Figure 15 shows higher diagonal proportions for nine of the ten foreground classes. The gains are most visible for pedestrian (0.30 to 0.39), people (0.18 to 0.27), motor (0.29 to 0.36), car (0.72 to 0.77), and bus (0.41 to 0.46). The raw count matrices support the same pattern: true positives increase from 16,253 to 18,679, while false negatives decrease from 19,717 to 17,050. This improvement is not uniform. Background false positives increase from 5908 to 7085, the tricycle diagonal decreases from 0.18 to 0.15, and van-to-car confusion remains 0.40. MF-YOLO therefore recovers more objects overall, but it does not reduce every class-specific error.

The image in Figure 16 contains 317 valid annotations: 280 small, 35 medium, and 2 large objects under the COCO area definitions. All responses were extracted from the best seed-0 checkpoints with the same input. Within MF-YOLO, GLSA suppresses broad high-intensity background responses while retaining localized responses around vehicles, road objects, and the dense region on the right; the fused P2 feature further preserves responses at dispersed small-object locations. This qualitative pattern is consistent with the gains in ${AP}_{S}$ and recall.

Figure 17 examines a dense traffic scene in which many vehicles and pedestrians occupy only a small region of the image. Compared with YOLO11n, MF-YOLO produces 16 additional true positives and 16 fewer false negatives without increasing the number of false positives. The result is consistent with better recovery of distant objects, although the 195 remaining false negatives show that severe crowding and limited object size continue to challenge the model.

Figure 18 provides a second crowded case with dense roadside pedestrians and closely spaced road users. MF-YOLO yields 23 more true positives and 23 fewer false negatives than YOLO11n, accompanied by one additional false positive. The comparison supports the recall improvement observed in the quantitative results, while also showing that occlusion, adjacent pedestrians, and visually similar categories still produce missed or incorrect detections.

### 5.10. Discussion and Limitations

Across the three matched seeds, adding GLSA increased mAP@0.5:0.95 by 0.204, 0.394, and 0.035 percentage points. The mean paired gain was 0.211 ± 0.180 percentage points, and the 95% confidence interval included zero. The direction was consistent, but the magnitude was small; GLSA should therefore be regarded as a modest refinement rather than the principal source of MF-YOLO’s improvement.

MF-YOLO provides its largest gains for the small and medium objects that dominate VisDrone, but this benefit comes with clear trade-offs. The parameter count falls by 13.2%, while GFLOPs rise from 6.323 to 14.365 and FP32 throughput falls from 144.22 to 70.50 FPS. AP for large objects also decreases. MF-YOLO is therefore suited to settings that prioritize small-object accuracy and can accommodate additional computation and weaker large-object performance.

The ablation results indicate that the P2 branch supplies the main single-component gain. Weighted bidirectional fusion is ineffective on its own but improves the model when combined with P2, while GLSA contributes a smaller increment. These findings are specific to the present dataset, training schedule, and implementation. Broader conclusions require evaluation on additional UAV datasets and deployment platforms.

## 6. Conclusions

This study presents MF-YOLO, a YOLO11n-based detector developed for small-object detection in UAV imagery. The model introduces a P2 prediction branch and connects the P2–P5 features through a 64-channel weighted bidirectional fusion path, with GLSA applied to C2–C5 before feature fusion. The ablation results clarify the contribution of each architectural component. The P2-only configuration improved small-object detection, whereas BiFPN-only did not produce a comparable benefit when used independently. Combining the two components gave better results than either individual configuration with seed 0. The additional improvement obtained from GLSA was smaller: the paired increase in mAP@0.5:0.95 was 0.211 ± 0.180 percentage points across three seeds. We therefore retained GLSA in the final A2 configuration but do not interpret this result as evidence of a statistically established gain. Compared with YOLO11n, MF-YOLO improved mean mAP@0.5 from 34.054% to 38.318% and mean mAP@0.5:0.95 from 19.964% to 22.658% on the validation split. Improvements were also observed on the held-out test-dev split. In the COCO-style evaluation, ${AP}_{S}$ and ${AP}_{M}$ increased by 4.102 and 2.354 percentage points, respectively, while ${AP}_{L}$ decreased by 8.190 percentage points. The improvement in small-object detection was accompanied by a clear computational cost. Although the parameter count fell by 13.2%, GFLOPs increased from 6.323 to 14.365, and FP32 batch-1 network-forward throughput decreased from 144.22 to 70.50 FPS on an RTX 4080 SUPER. MF-YOLO is therefore most appropriate for UAV applications in which small-object detection accuracy is more important than computational efficiency or large-object performance.

Several limitations remain to be addressed. The most immediate priorities are to reduce the computation introduced by the P2 pathway and to recover part of the lost large-object performance. A more selective fusion route could adjust the contribution of high-resolution features according to object scale or scene content rather than propagating them uniformly through the pyramid. Lightweight channel allocation and model compression may further improve deployment efficiency. The model should also be evaluated on additional UAV datasets and embedded platforms to determine whether the present improvements remain consistent at different scene densities, flight altitudes, and hardware constraints.

## Figures and Tables

**Figure 1:**
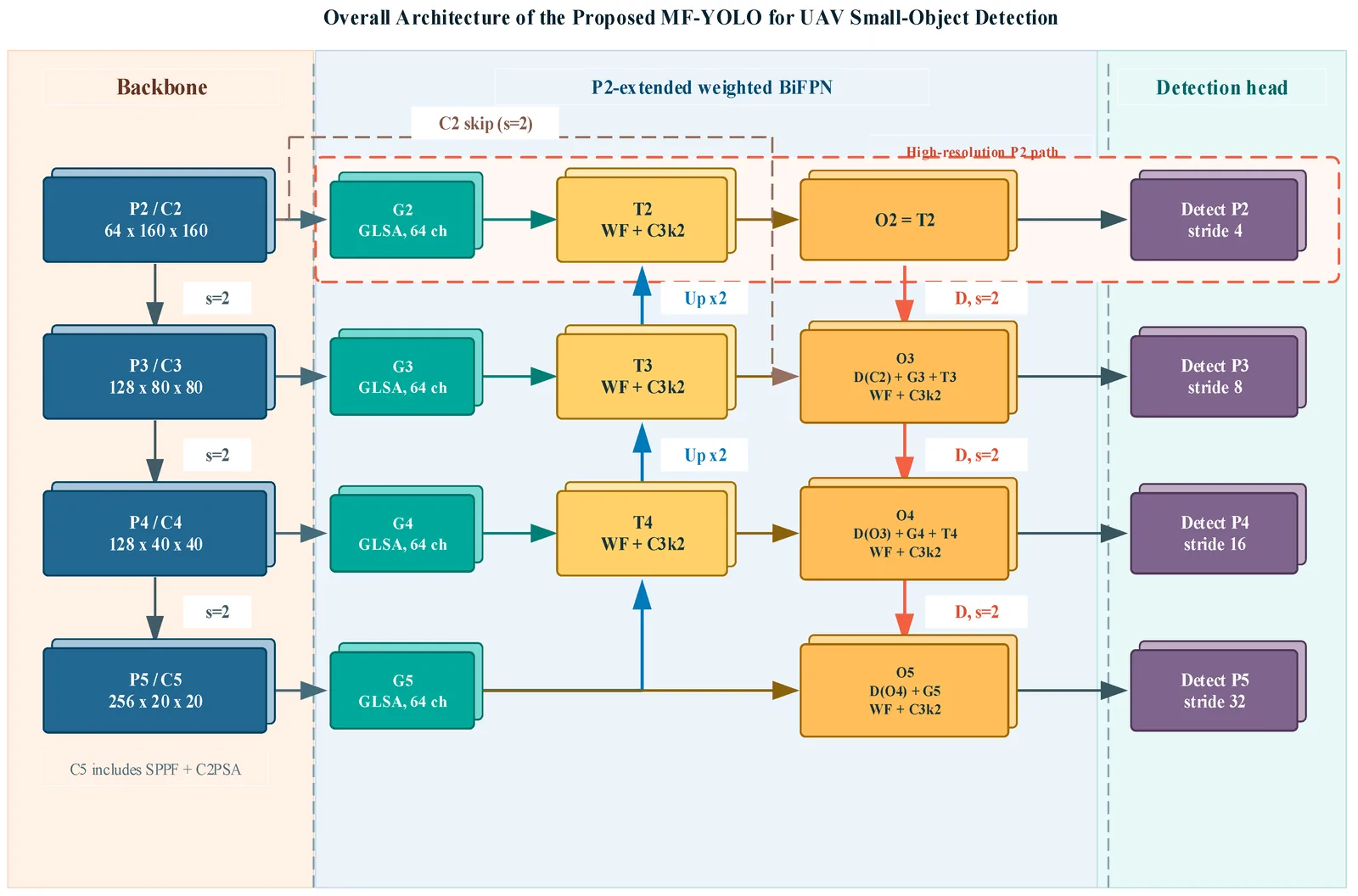
Overall architecture of the final MF-YOLO detector.

**Figure 2:**
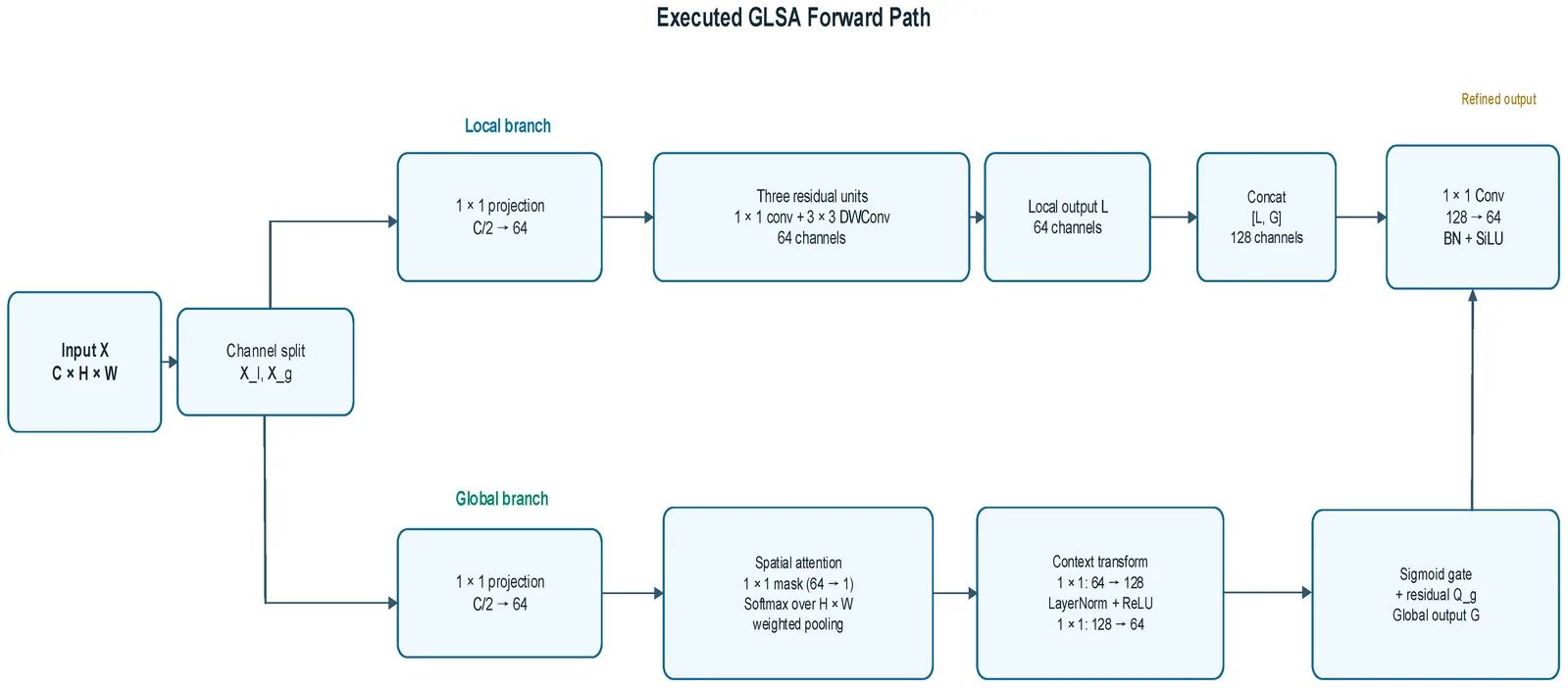
Executed forward path of the GLSA module.

**Figure 3:**
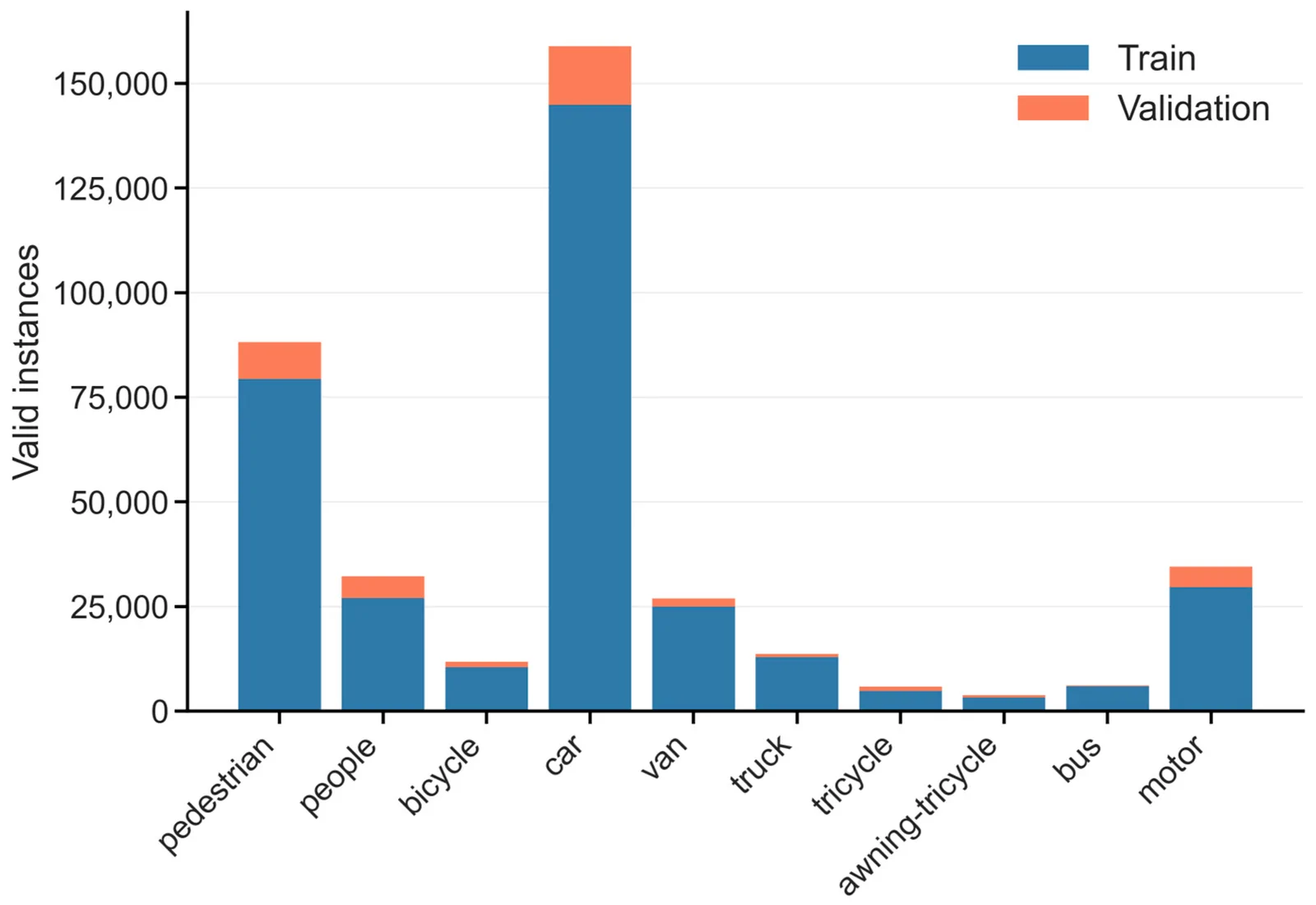
Class distribution in VisDrone2019-DET.

**Figure 4:**
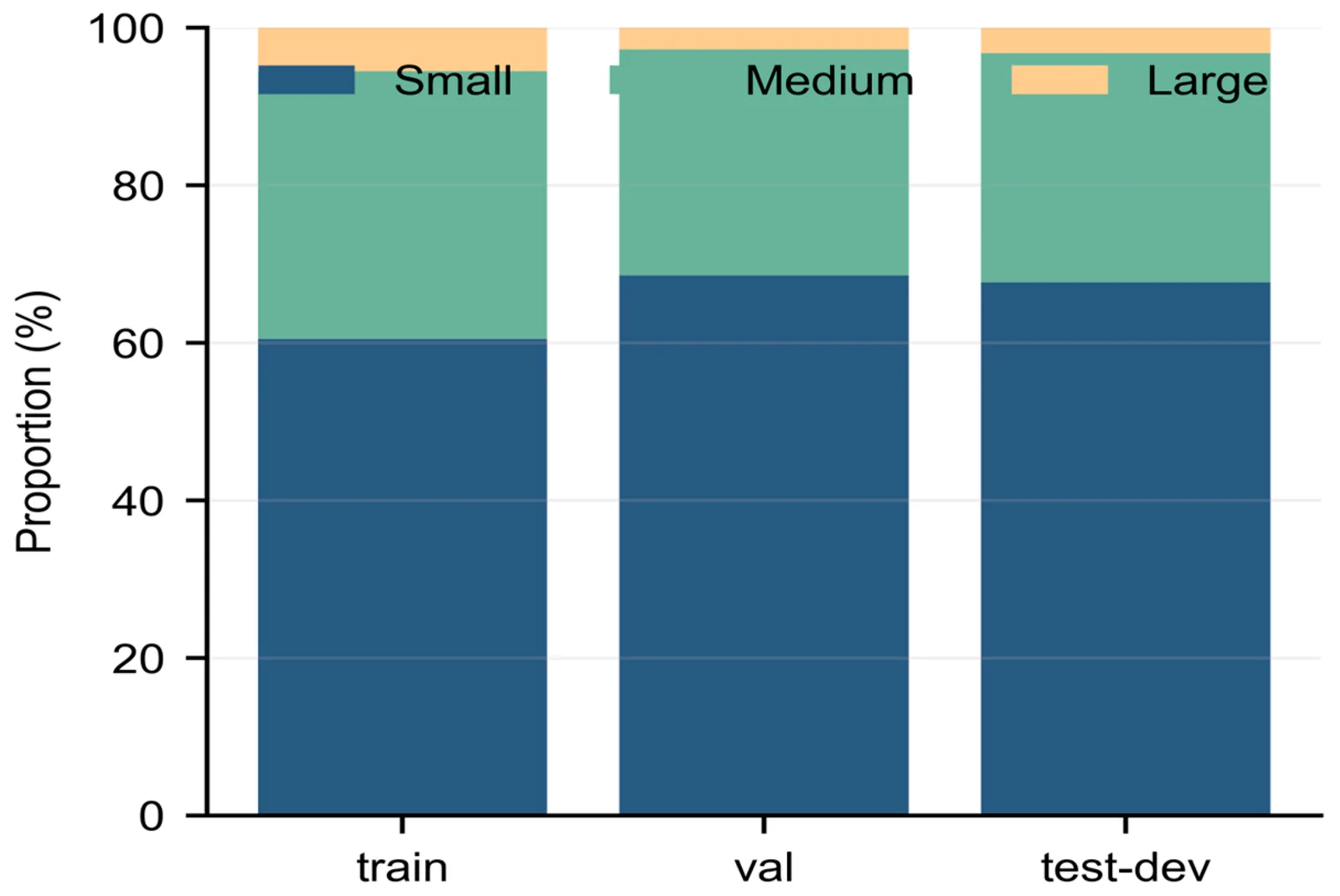
COCO-style object-size distribution in VisDrone2019-DET. Blue, green, and yellow denote small, medium, and large objects, respectively.

**Figure 5:**
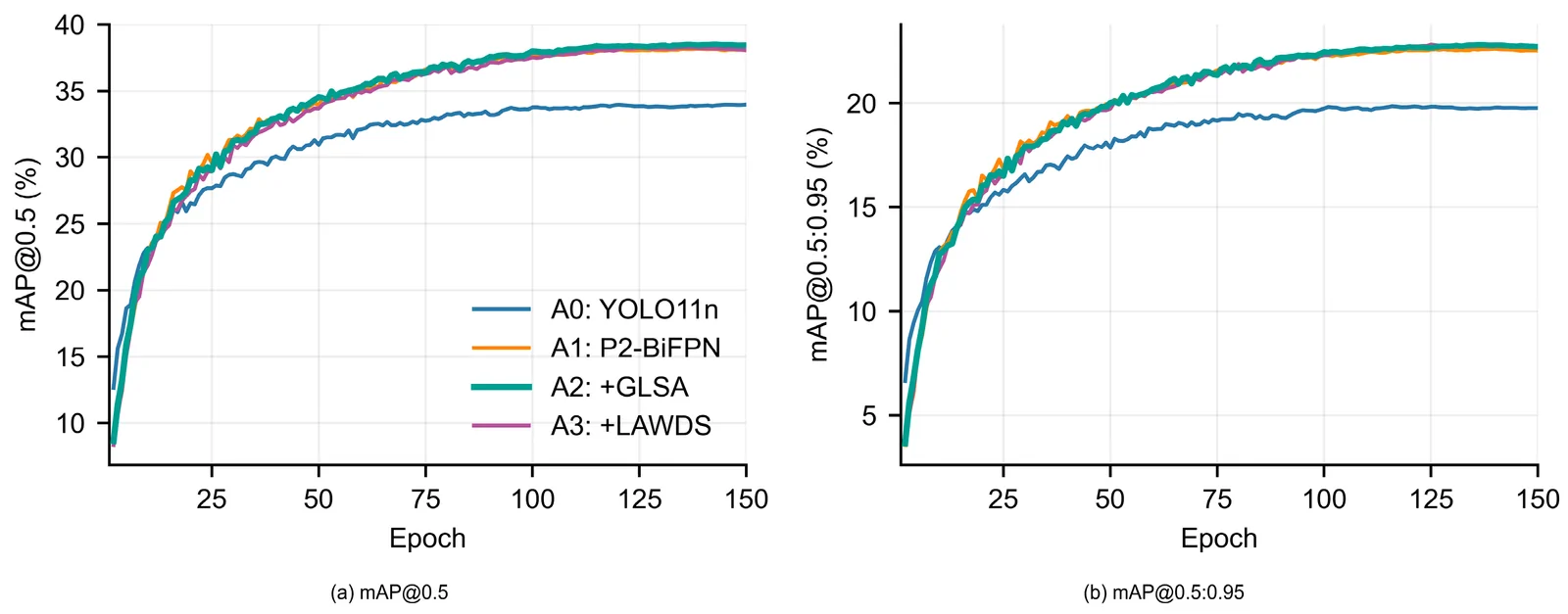
Seed-0 validation trajectories for the progressive A0–A3 architecture sequence: (**a**) mAP@0.5; (**b**) mAP@0.5:0.95.

**Figure 6:**
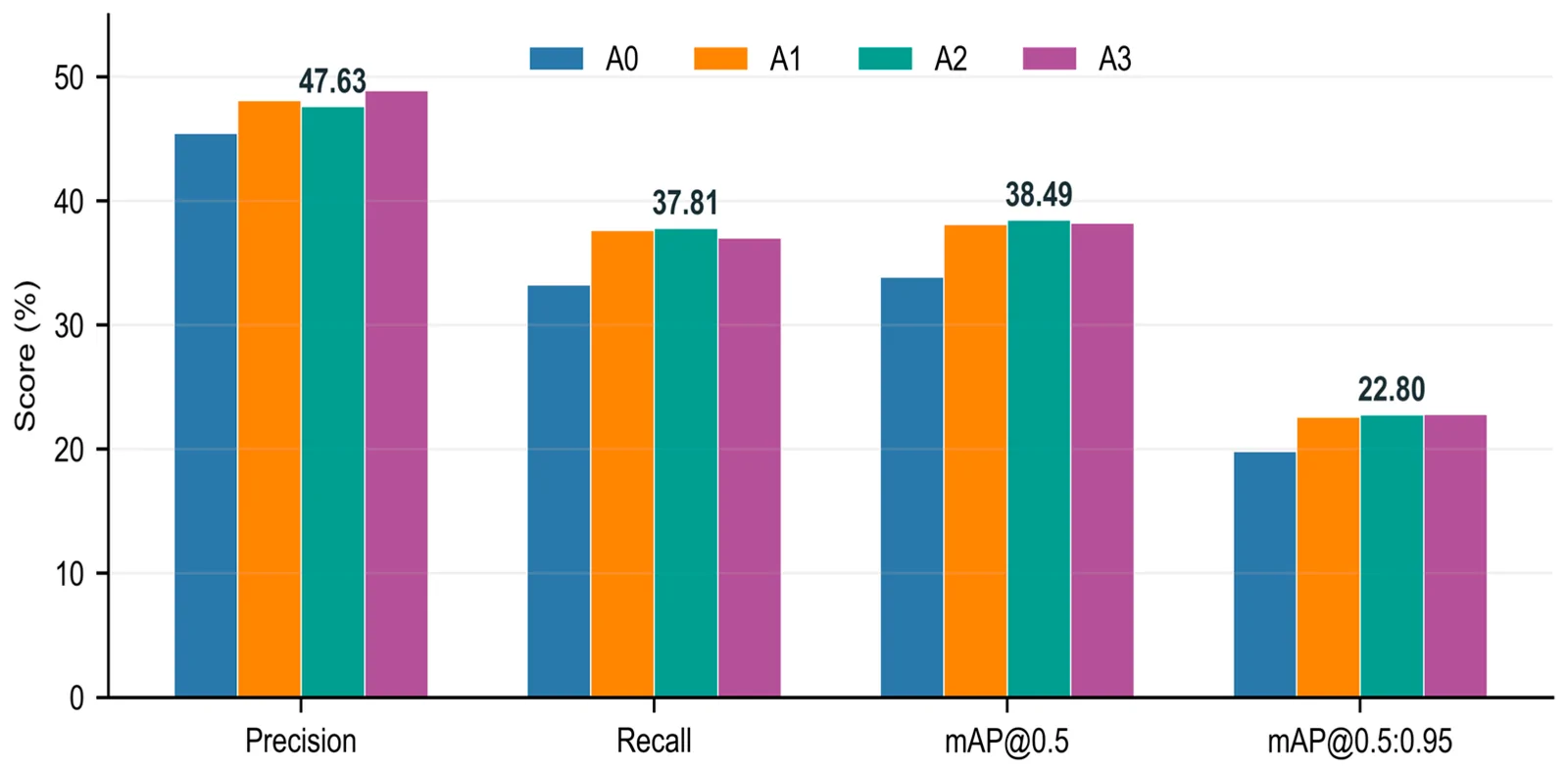
Seed-0 precision, recall, and AP comparison; numeric labels identify the selected A2 configuration.

**Figure 7:**
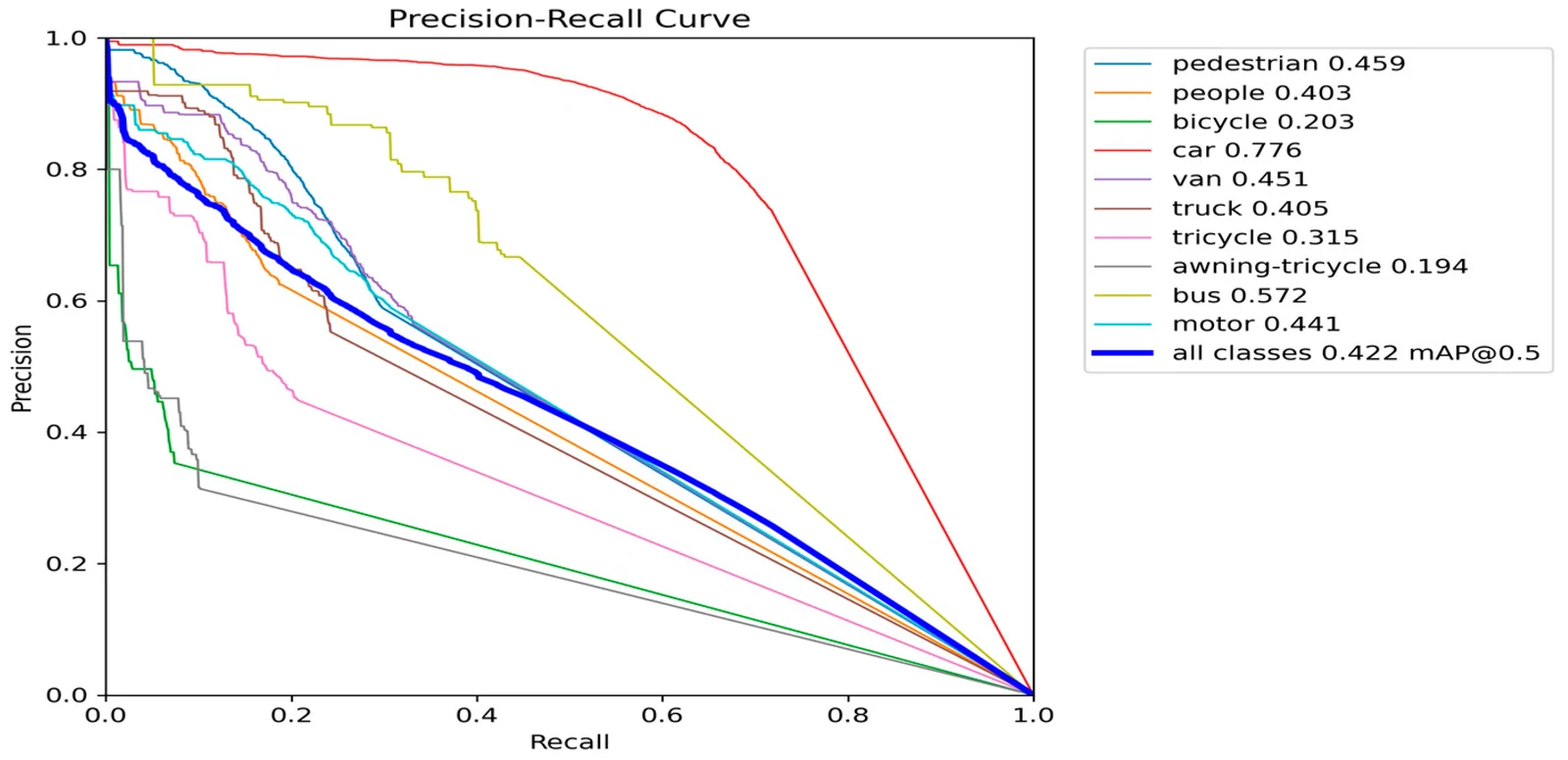
Class-wise and macro-average precision–recall curves for YOLO11n (A0) on the VisDrone2019-DET validation split using seed 0.

**Figure 8:**
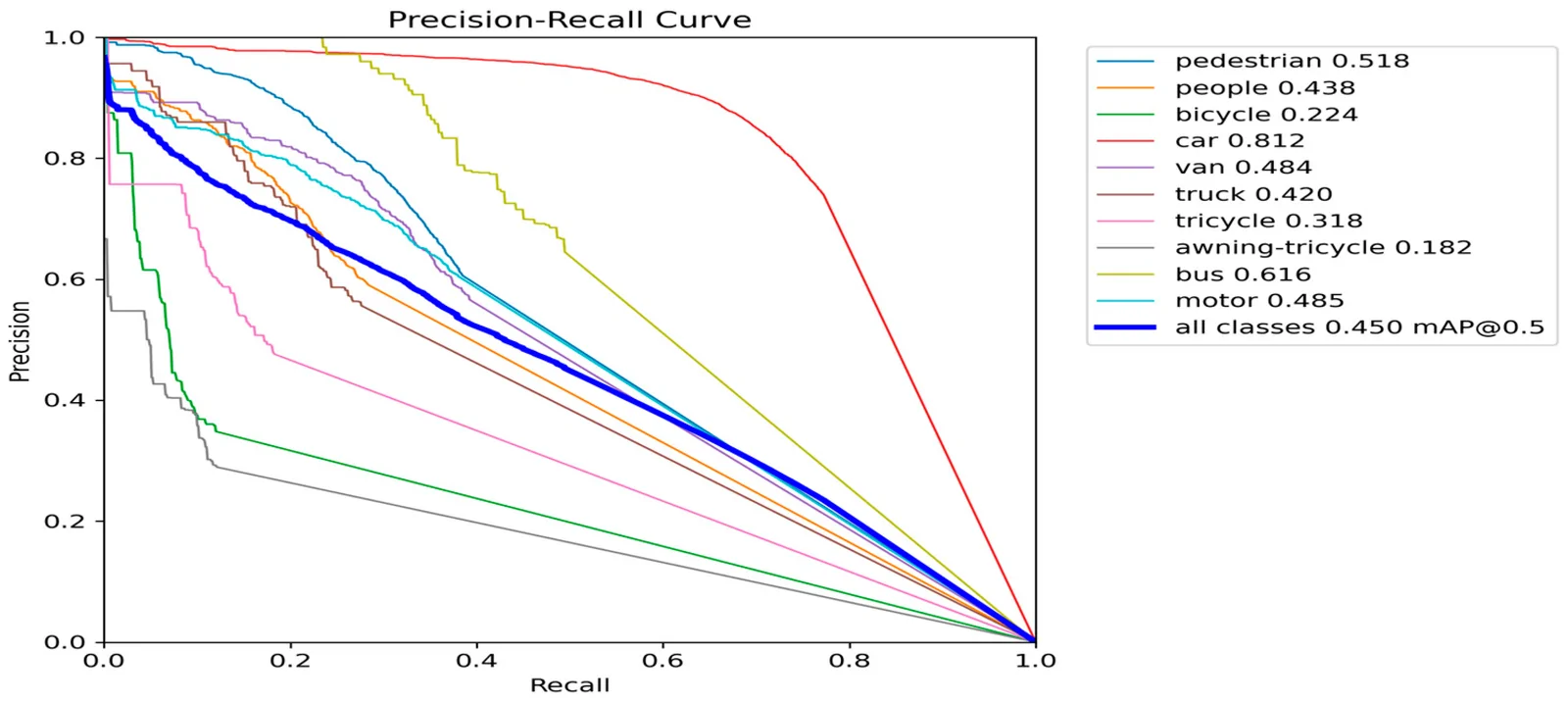
Class-wise and macro-average precision–recall curves for MF-YOLO (A2) on the VisDrone2019-DET validation split using seed 0.

**Figure 9:**
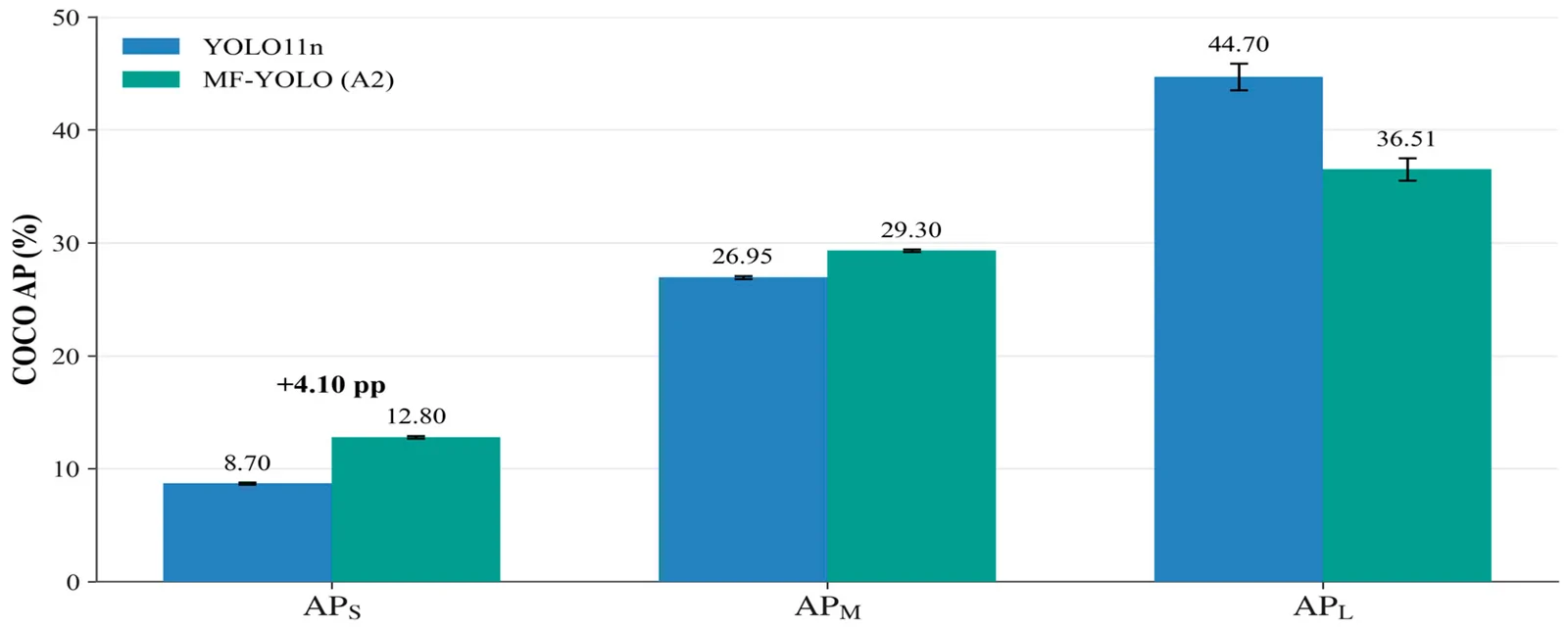
COCO-style AP by object scale for YOLO11n and MF-YOLO (A2).

**Figure 10:**
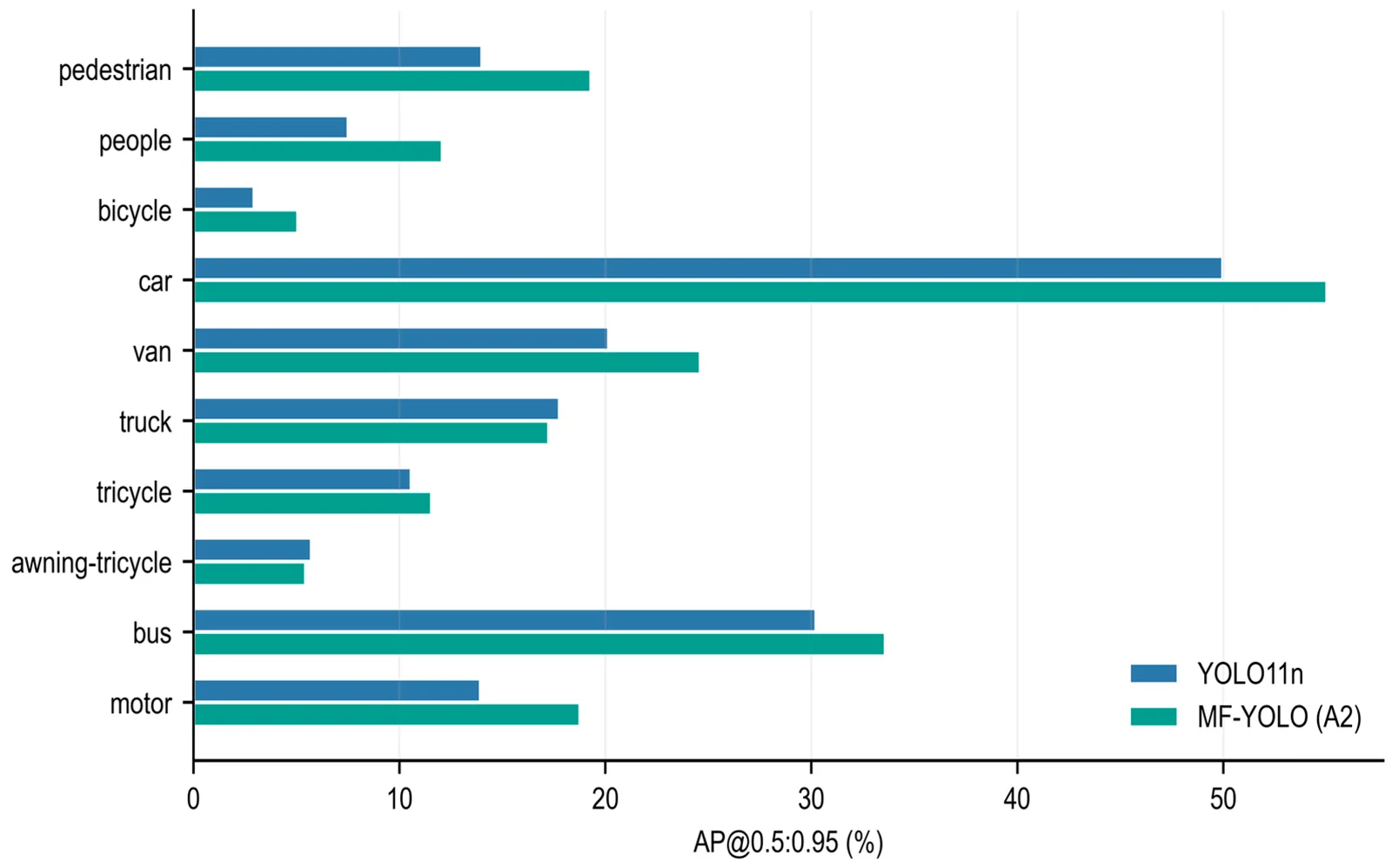
Per-class AP@0.5:0.95 under the COCO-style evaluation protocol.

**Figure 11:**
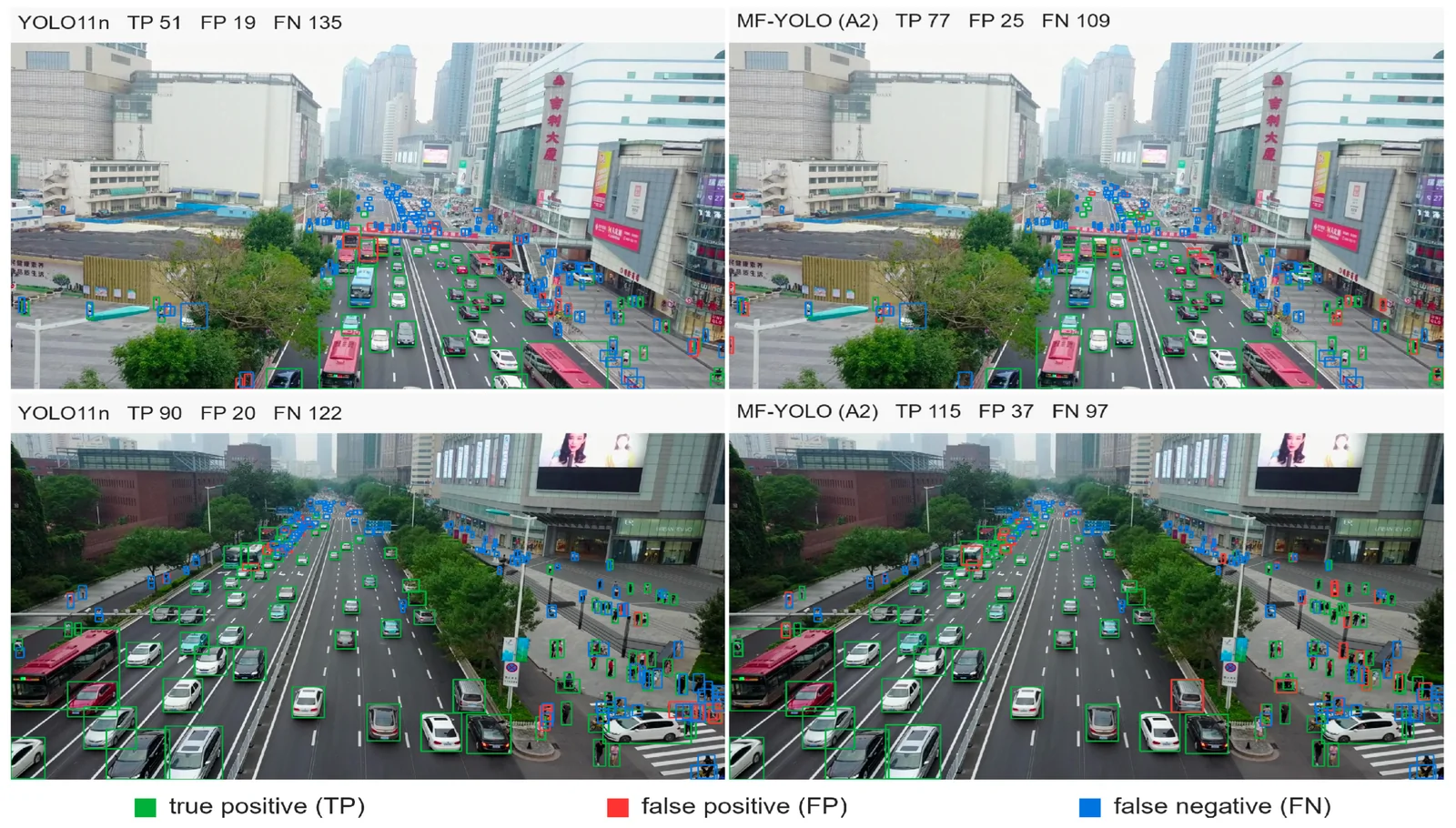
Qualitative comparison on two scenes from the publicly available VisDrone2019-DET validation set [1], with prediction and error overlays generated by the authors. Green denotes matched detections, red denotes unmatched predictions, and blue denotes missed ground-truth objects.

**Figure 12:**
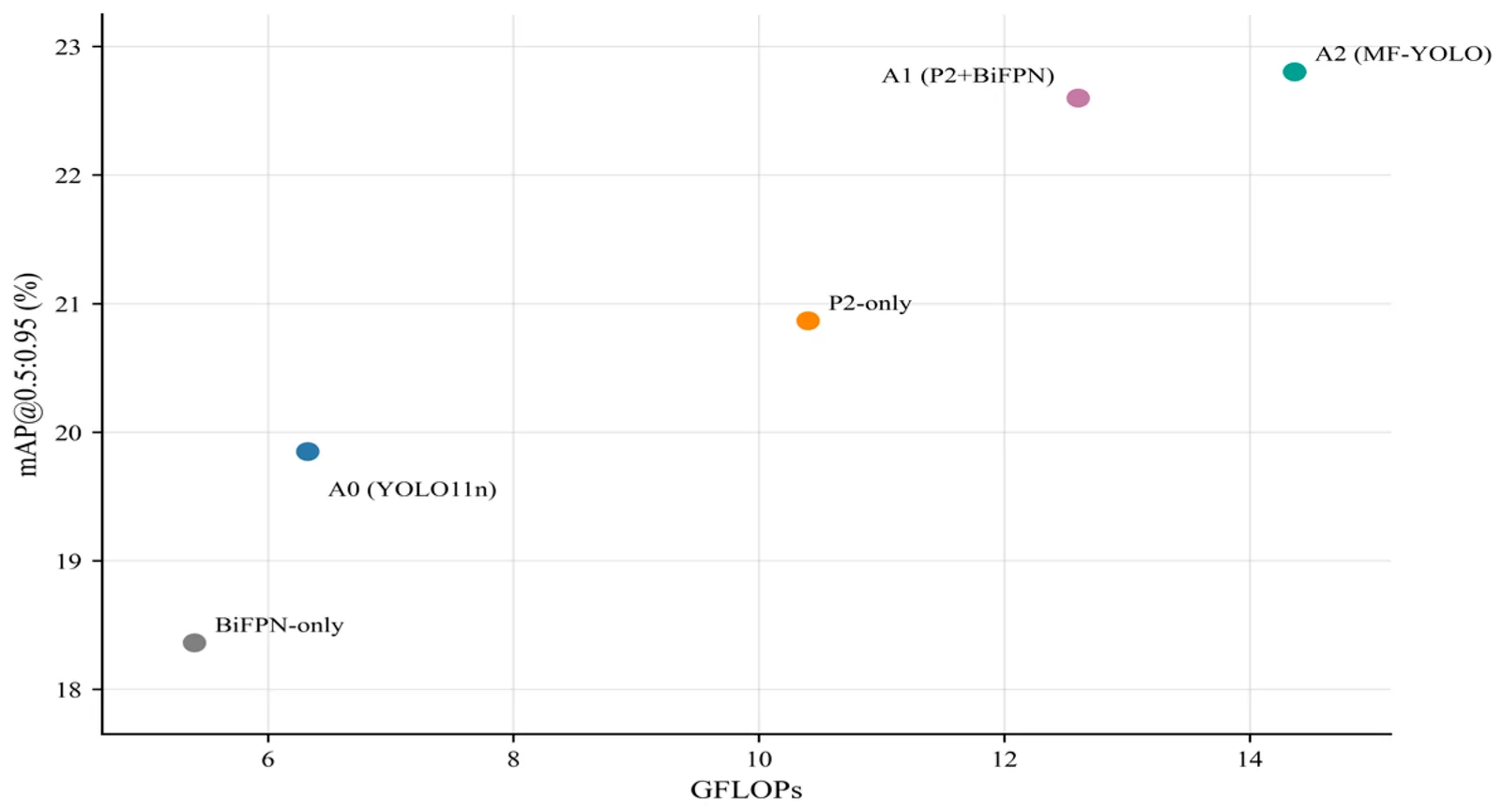
Accuracy–complexity trade-off among the five seed-0 configurations.

**Figure 13:**
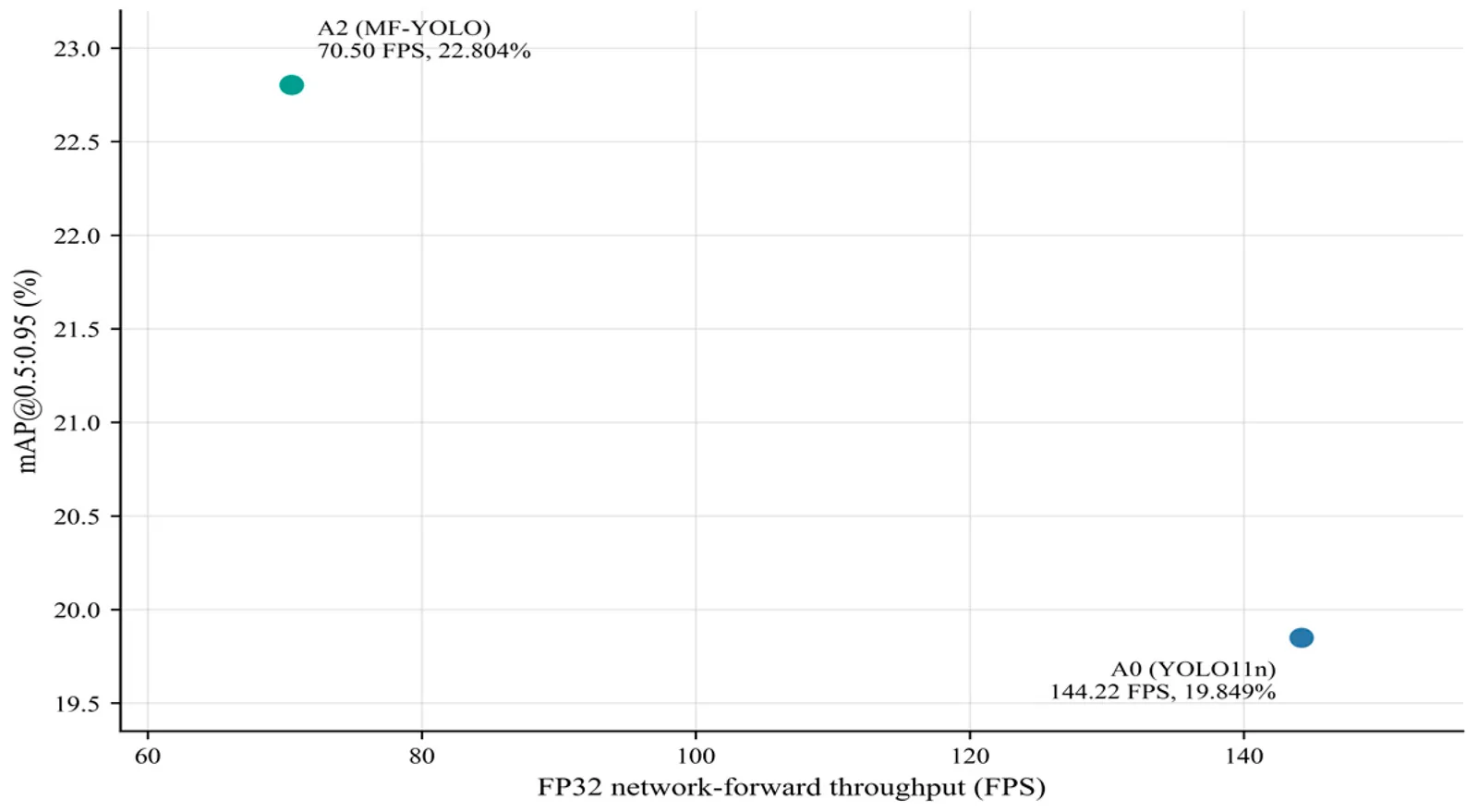
Accuracy–throughput comparison of YOLO11n (A0) and MF-YOLO (A2).

**Figure 14:**
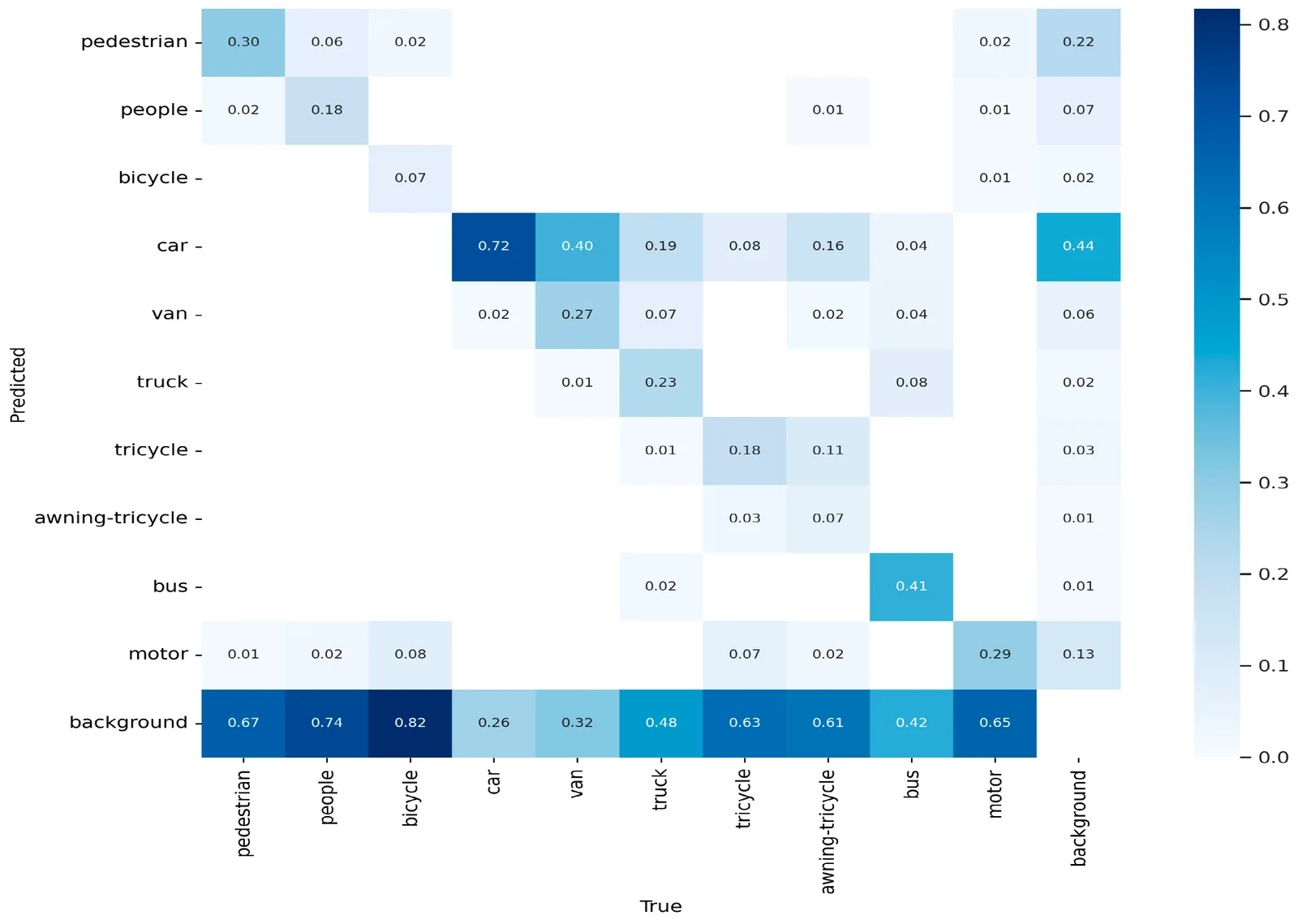
Normalized confusion matrix for YOLO11n (A0) on the VisDrone2019-DET validation split.

**Figure 15:**
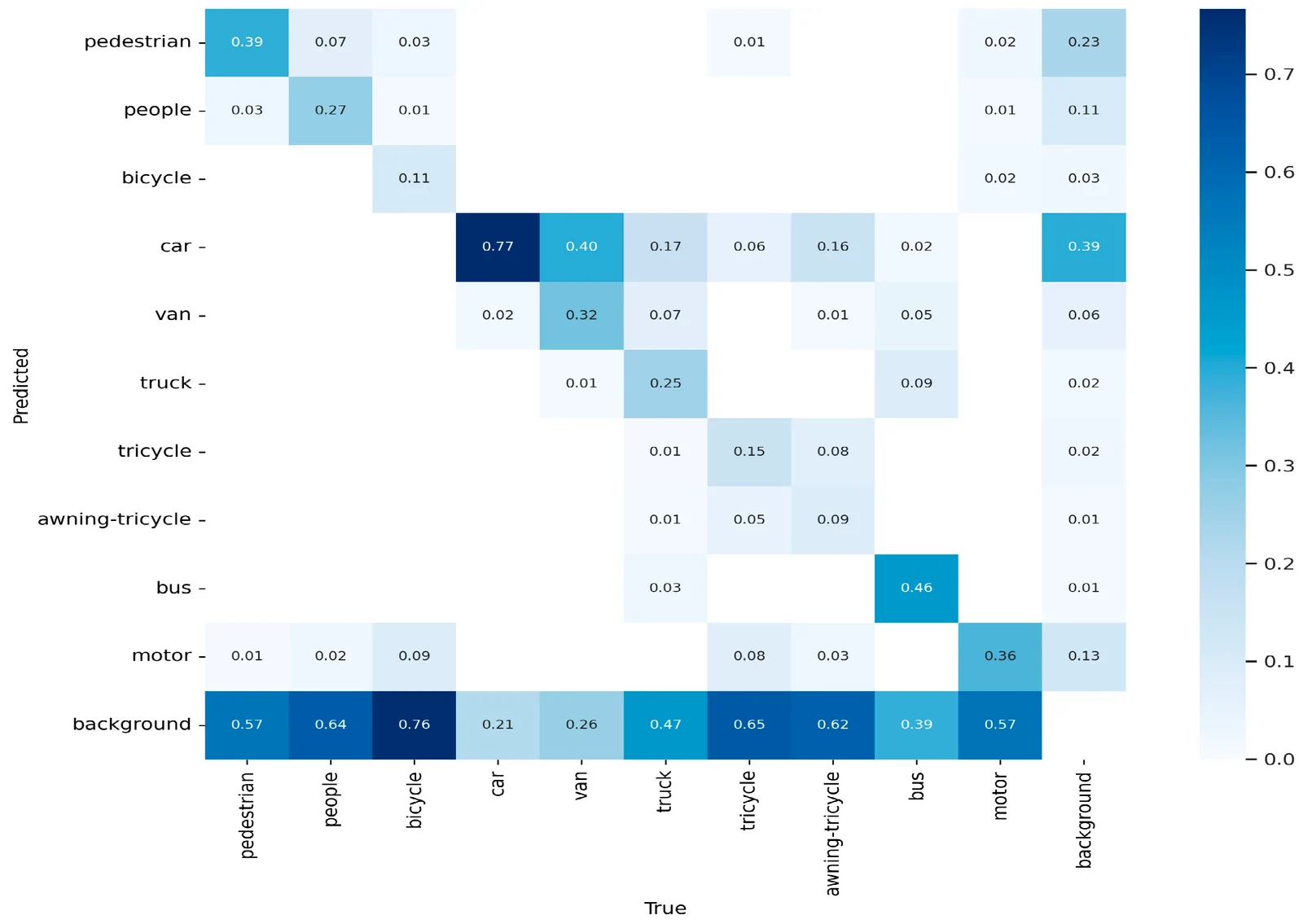
Normalized confusion matrix for MF-YOLO (A2) on the VisDrone2019-DET validation split.

**Figure 16:**
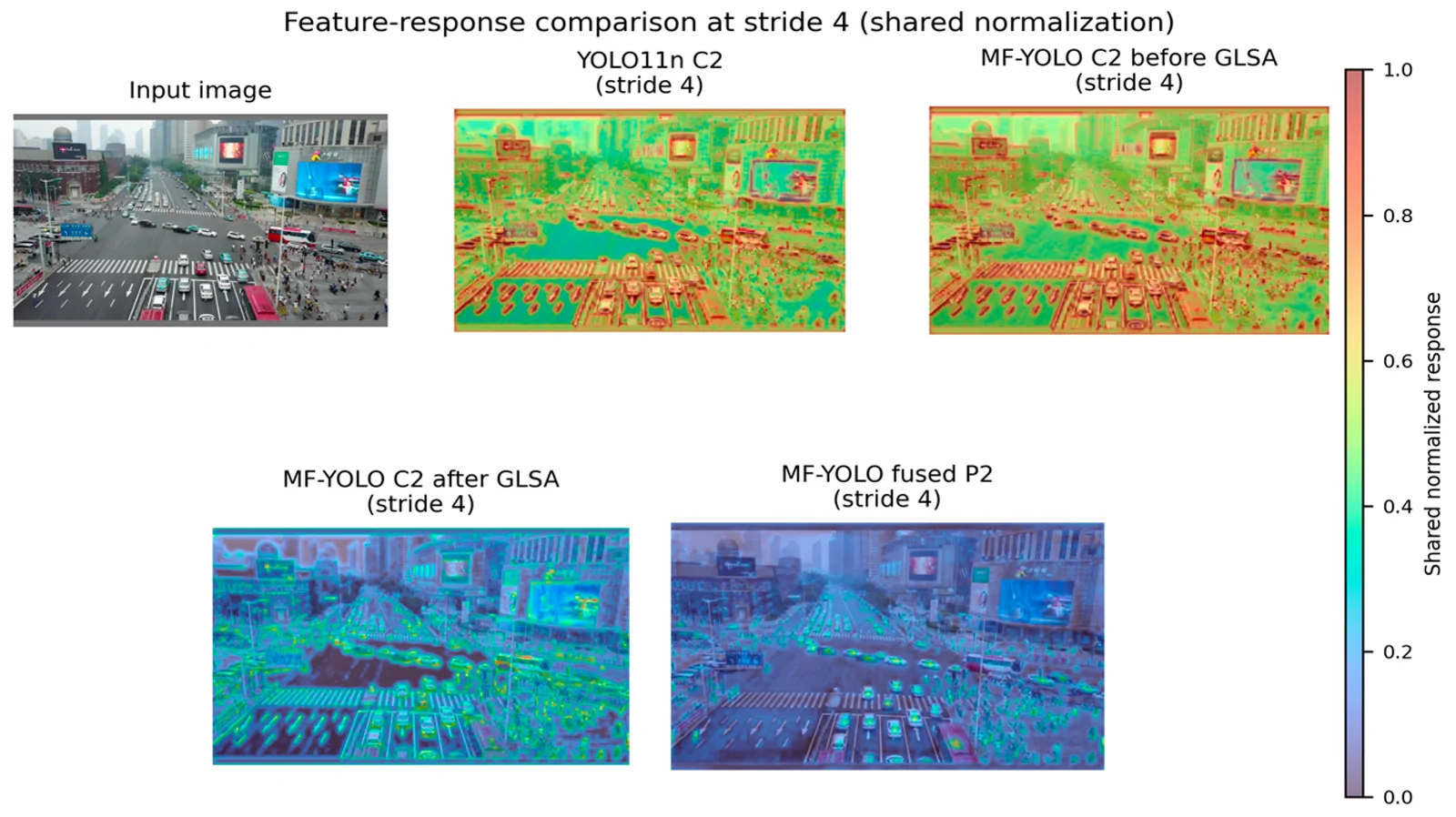
Input image and matched feature responses from YOLO11n C2, MF-YOLO C2 before and after GLSA, and the fused P2 feature.

**Figure 17:**
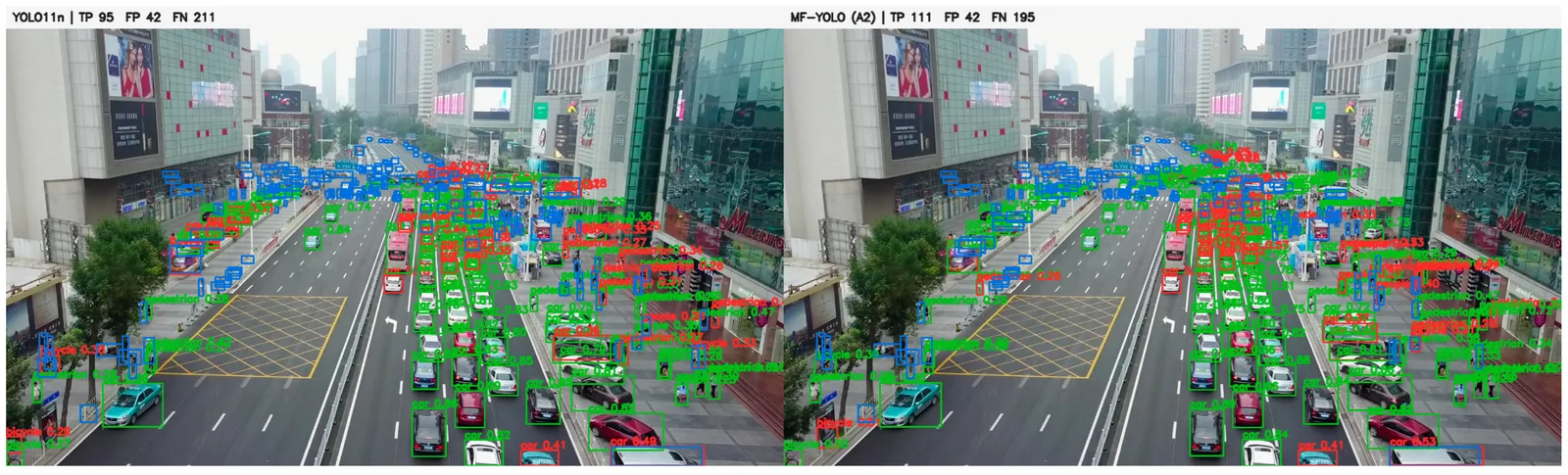
Qualitative comparison in a dense long-range traffic scene. The left and right panels show YOLO11n and MF-YOLO (A2), respectively. Green boxes denote true positives (TP), red boxes denote false positives (FP), and blue boxes denote false negatives (FN); the text above each panel reports the corresponding counts.

**Figure 18:**
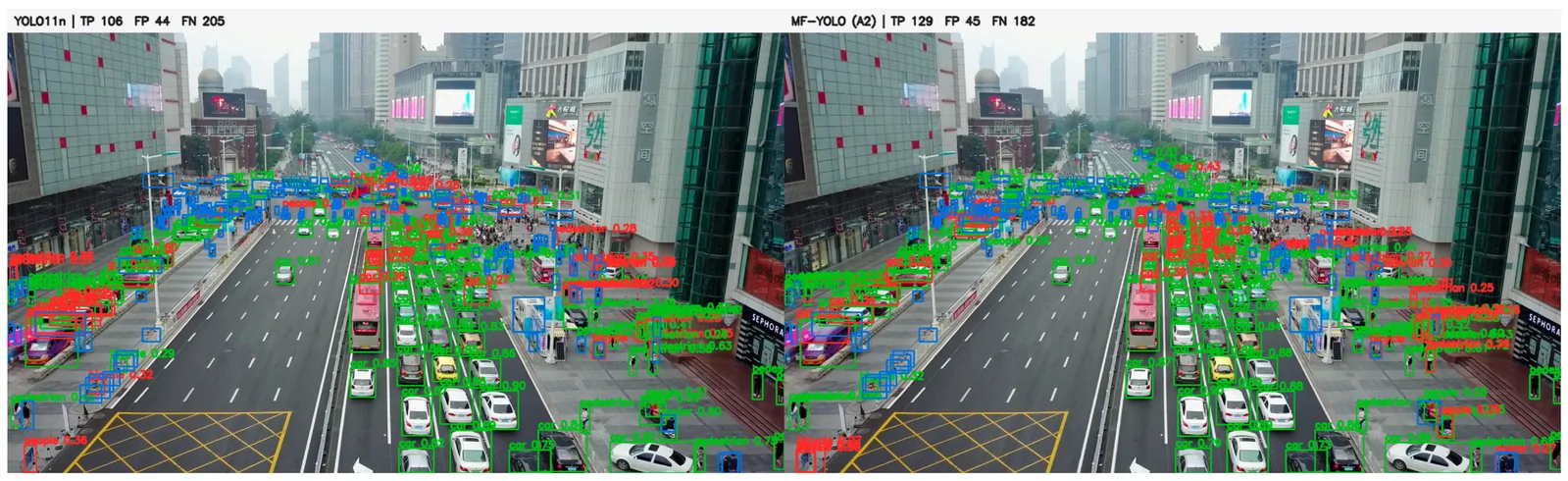
Qualitative comparison in a scene with dense roadside pedestrians and class ambiguity. The left and right panels show YOLO11n and MF-YOLO (A2), respectively. Green boxes denote true positives (TP), red boxes denote false positives (FP), and blue boxes denote false negatives (FN); the text above each panel reports the corresponding counts.

**Table 1:** Architectural comparison between MF-YOLO and the closest existing designs.

Method	Base Task	High-Resolution Prediction	Fusion or Refinement Strategy	Key Differences from MF-YOLO
EfficientDet	General-purpose object detection	P3-P7, no P2 predictions	Repeatedly weighted BiFPN	Conceptual source of weighted BiFPN; no pre-fusion GLSA at C2–C5 or four-scale P2–P5 detection path
DuAT	Medical image segmentation	Does not use YOLO detection heads	Employs GLSA	Conceptual source of GLSA; differs in task, output head, and cross-scale information path
HSP-YOLOv8	Drone detection	Adds a branch for detecting small objects	SPD-Conv and Soft-NMS	Does not employ GLSA prior to C2–C5 fusion or the weighted P2–P5 path described in this paper
SOD-YOLO	Drone detection	Three-scale outputs (P2–P4)	BSSI-FPN, DySample and RFCBAM	Differences in prediction scales and feature fusion methods
SRTSOD-YOLO	Drone detection	Introduces P2 and removes P5	MFCAM, GAC-FPN and an improved loss function	Uses P2–P4; MF-YOLO retains P2–P5 and applies GLSA to each scale separately prior to fusion
MF-YOLO	Drone detection	Four-scale output: P2, P3, P4, P5	GLSA prior to C2–C5 fusion; 64-channel weighted bidirectional path	Contribution is limited to task-specific organization, module placement, and controlled validation

**Table 2:** Structural comparison between YOLO11n and MF-YOLO.

Property	YOLO11n	MF-YOLO (A2)
Prediction scales	P3, P4, P5	P2, P3, P4, P5
Finest prediction stride	8	4
Pre-fusion enhancement	None	GLSA at C2-C5
Cross-scale operation	Three-scale concatenation path	Learnable weighted bidirectional fusion
Aligned neck width	Not fixed across all inputs	64 channels
Direct high-resolution shortcut	None	Projected C2 input to the P3 bottom-up node

**Table 3:** Audited VisDrone2019-DET split statistics.

Split	Images	Valid Boxes	Ignored Regions	Small	Medium	Large
Train	6471	343,204	10,345	207,524	116,696	18,984
Validation	548	38,759	1410	26,575	11,116	1068
Test-dev	1610	75,102	2445	50,816	21,876	2410

**Table 4:** Unified training configuration.

Item	Setting	Item	Setting
Input size	640 × 640	Epochs	150
Physical batch size	8	Optimizer	SGD
Initial learning rate	0.01	Final LR multiplier	0.01
Momentum	0.937	Weight decay	0.0005
Warm-up epochs	3	AMP	Enabled
HSV gains (H/S/V)	0.015/0.7/0.4	Translation/scale	0.1/0.5
Horizontal flip	0.5	Mosaic	1.0; closed in final 10 epochs
Workers	2	Maximum detections	300

**Table 10:** Three-seed evaluation on the held-out VisDrone2019-DET test-dev split.

Model	P Mean ± SD (%)	R Mean ± SD (%)	mAP@0.5 Mean ± SD (%)	mAP@0.5:0.95 Mean ± SD (%)
YOLO11n	40.121 ± 0.473	30.575 ± 0.131	28.271 ± 0.132	15.898 ± 0.109
MF-YOLO	42.117 ± 0.579	33.002 ± 0.250	31.127 ± 0.358	17.342 ± 0.240
Difference(pp)	+1.996	+2.427	+2.856	+1.444

**Table 11:** Three-seed COCO-style scale-specific AP on the validation split.

Model	AP@0.5:0.95 (%)	AP@0.5 (%)	${AP}_{S}$ (%)	${AP}_{M}$ (%)	${AP}_{L}$ (%)
YOLO11n	17.276 ± 0.136	29.316 ± 0.192	8.700 ± 0.092	26.950 ± 0.132	44.697 ± 1.172
MF-YOLO	20.260 ± 0.112	34.804 ± 0.169	12.802 ± 0.115	29.304 ± 0.108	36.507 ± 0.995
Difference(pp)	+2.984	+5.488	+4.102	+2.354	−8.190

**Table 12:** .Object-scale-specific AP results for the component-ablation configurations (seed 0).

Configuration	P2 Branch	Weighted BiFPN	GLSA	${AP}_{S}$ (%)	${AP}_{M}$ (%)	${AP}_{L}$ (%)	Δ${AP}_{S}$	Δ${AP}_{L}$
A0(YOLO11n)	No	No	No	8.624	26.945	43.475	-	-
P2-only	Yes	No	No	11.145	26.804	37.154	+2.521	−6.321
BiFPN-only	No	Yes	No	7.430	25.098	37.071	−1.194	−6.404
A1	Yes	Yes	No	12.554	29.129	37.253	+3.930	−6.222
A2	Yes	Yes	Yes	12.792	29.240	37.629	+4.168	−5.846

**Table 13:** Mean per-class AP@0.5:0.95 across three seeds.

Class	YOLO11n (%)	MF-YOLO (%)	Difference (pp)
Pedestrian	13.995	19.284	+5.289
People	7.504	12.067	+4.563
Bicycle	2.936	5.054	+2.118
Car	49.969	55.014	+5.045
Van	20.150	24.603	+4.453
Truck	17.761	17.245	–0.516
Tricycle	10.574	11.552	+0.978
Awning-tricycle	5.721	5.440	–0.281
Bus	30.222	33.581	+3.360
Motor	13.934	18.758	+4.824

**Table 14:** Comparison of modern YOLO baselines using harmonized experimental settings (seed 0).

Model	P (%)	R (%)	mAP@0.5 (%)	mAP@0.5:0.95 (%)	Parameters (M)	GFLOPs
YOLOv8n	43.765	33.987	33.585	19.645	2.692	7.0
YOLOv10n	45.012	35.369	35.369	20.531	2.711	8.4
YOLO11n	45.471	33.277	33.897	19.849	2.584	6.323
MF-YOLO	47.630	37.814	38.490	22.804	2.244	14.365

**Table 15:** Literature-reported comparisons on the VisDrone2019 validation split.

Method	Base Model	Epochs	mAP@0.5 (%)	mAP@0.5:0.95 (%)	Parameters (M)	GFLOPs	Source and Protocol
HSP-YOLOv8	YOLOv8s	200	49.6	32.9	11.5	50.0	Original paper; 640 × 640; 200 epochs; SGD
SOD-YOLO-n	YOLOv8	150	33.0	19.3	0.60	7.8	Original paper; 640 × 640; 150 epochs; SGD; no pretrained weights
TOE-YOLO	YOLO11n	200	33.8	19.7	2.53	6.6	Original paper; 640 × 640; 200 epochs; Adam
SRTSOD-YOLO-n	YOLO11n	300	36.3	21.8	3.50	7.4	Original paper; 640 × 640; 300 epochs; three random seeds
MF-YOLO	YOLO11n	150	38.318 ± 0.378	22.658 ± 0.239	2.244	14.365	This study; 640 × 640; 150 epochs; three random seeds

**Table 16:** Complexity and network-forward throughput of YOLO11n and MF-YOLO for the controlled hardware benchmark.

Model	Precision Mode	Parameters (M)	GFLOPs	Median Latency (Ms)	Fps	Peak Gpu Memory (Mib)
YOLO11n	FP32	2.584	6.323	6.934	144.22	113.9
YOLO11n	FP16	2.584	6.323	7.258	137.79	111.2
MF-YOLO	FP32	2.244	14.365	14.184	70.50	196.2
MF-YOLO	FP16	2.244	14.365	14.844	67.37	156.0

## Data Availability

The VisDrone2019-DET dataset used in this study is publicly available from the VisDrone project. The processed audit records, training configurations, and experimental logs supporting the reported results are available from the corresponding author upon reasonable request.
